# Potential impacts on ecosystem services of land use transitions to second‐generation bioenergy crops in GB

**DOI:** 10.1111/gcbb.12263

**Published:** 2015-06-08

**Authors:** Suzanne Milner, Robert A. Holland, Andrew Lovett, Gilla Sunnenberg, Astley Hastings, Pete Smith, Shifeng Wang, Gail Taylor

**Affiliations:** ^1^ Centre for Biological SciencesUniversity of SouthamptonSouthamptonSO17 1BJUK; ^2^ School of Environmental SciencesUniversity of East AngliaNorwichNR4 7TJUK; ^3^ Institute of Biological and Environmental SciencesUniversity of Aberdeen23 St Machar DriveAberdeenAB24 3UUUK

**Keywords:** biofuel crops, ecological processes, ecosystem services, GIS, land use, *Miscanthus*, short‐rotation coppice, short‐rotation forestry, sustainability, trade‐offs

## Abstract

We present the first assessment of the impact of land use change (LUC) to second‐generation (2G) bioenergy crops on ecosystem services (ES) resolved spatially for Great Britain (GB). A systematic approach was used to assess available evidence on the impacts of LUC from arable, semi‐improved grassland or woodland/forest, to 2G bioenergy crops, for which a quantitative ‘threat matrix’ was developed. The threat matrix was used to estimate potential impacts of transitions to either *Miscanthus*, short‐rotation coppice (SRC, willow and poplar) or short‐rotation forestry (SRF). The ES effects were found to be largely dependent on previous land uses rather than the choice of 2G crop when assessing the technical potential of available biomass with a transition from arable crops resulting in the most positive effect on ES. Combining these data with constraint masks and available land for SRC and *Miscanthus* (SRF omitted from this stage due to lack of data), south‐west and north‐west England were identified as areas where *Miscanthus* and SRC could be grown, respectively, with favourable combinations of economic viability, carbon sequestration, high yield and positive ES benefits. This study also suggests that not all prospective planting of *Miscanthus* and SRC can be allocated to agricultural land class (ALC) ALC 3 and ALC 4 and suitable areas of ALC 5 are only minimally available. Beneficial impacts were found on 146 583 and 71 890 ha when planting *Miscanthus* or SRC, respectively, under baseline planting conditions rising to 293 247 and 91 318 ha, respectively, under 2020 planting scenarios. The results provide an insight into the interplay between land availability, original land uses, bioenergy crop type and yield in determining overall positive or negative impacts of bioenergy cropping on ecosystems services and go some way towards developing a framework for quantifying wider ES impacts of this important LUC.

## Introduction

Public concern that bioenergy crops will encroach on land needed for food and animal feed is increasing (Rathmann *et al*., 2010; Tirado *et al*., 2010; Valentine *et al*., 2012), despite the fact that in the United Kingdom, only 1.8% of agricultural land was used for bioenergy feedstock production in 2010 (DEFRA, 2013) and 4% of agricultural land is unutilized (DEFRA, 2013). In Great Britain (GB), there are approximately 22.9 M ha of land in total (Lovett *et al*., 2014). Of this land, there is approximately 17.5 M ha that is suitable for planting, that is with an Agricultural Land Classification (ALC) other than nonagricultural and urban areas. This suggests there is a large potential area for crop growth. Alongside these concerns, climate change and population increase are placing additional pressure on land to deliver food, water and energy (Godfray *et al*., 2010), while maintaining a range of ecosystem services (ES) (Manning *et al*., 2014). Population increase, with additional urbanization of agricultural land, will also impact negatively on the delivery of ES as identified by Eigenbrod *et al*. (2011).

The impact of growing bioenergy and biofuel feedstock crops has been of particular concern, with some suggesting the greenhouse gas (GHG) balance of food crops used for ethanol and biodiesel may be no better or worse than fossil fuels (Fargione *et al*., 2008; Searchinger *et al*., 2008). This is controversial, as the allocation of GHG emissions to the management and the use of coproducts can have a large effect on the total carbon footprint of resulting bioenergy products (Whitaker *et al*., 2010; Davis *et al*., 2013). The potential consequences of land use change (LUC) to bioenergy on GHG balance through food crop displacement or ‘indirect’ land use change (iLUC) are also an important consideration (Searchinger *et al*., 2008). As a consequence, much effort is now focussed on determining the GHG balance of bioenergy cropping systems, but rather, less research has been undertaken on the impacts of bioenergy cropping on a wider range of ES (Donnelly *et al*., 2011). This is an important omission, as rapid changes are currently occurring in the policy landscape.

UK policy has recently been changed to reduce first‐generation (food crop feedstock)‐based bioenergy production (European Commission, 2012). Also, the minimum required GHG savings threshold for bioenergy is increasing, and an iLUC factor will be incorporated to account for carbon emissions (Searchinger *et al*., 2008; Plevin *et al*., 2010; Arima *et al*., 2011). There is also a general statement in the proposed directive that land of high biodiversity value should not be used for bioenergy cropping, but at a time when further sustainable intensification will be required – ‘getting more from less’ – this seems inadequate for landscape‐scale management of the environment, with respect to crop types and their usage. A focus on only GHG balance and biodiversity ignores a range of other ES such as water quality, where evidence‐based policy development is required for land use decisions, which is currently lacking (Bateman *et al*., 2013).

It has been proposed that second‐generation (2G) bioenergy and biofuel feedstocks can provide part of the solution to this issue, as they may be grown on land that is of poorer quality and more marginal areas than those required for food production (Hastings *et al*., 2009a,b; Tilman *et al*., 2009; Valentine *et al*., 2012). 2G feedstocks are defined here as perennial, lignocellulosic feedstocks that are nonfood crops (Valentine *et al*., 2012). In temperate climates, these 2G crops are likely to be *Miscanthus*, and fast‐growing trees such as poplar and willow as short‐rotation coppice (SRC) or poplar as short‐rotation forestry (SRF) (Hastings *et al*., 2014). Aylott *et al*. (2010) identified 0.8 Mha of land in England that could produce 7.5 Mt of SRC biomass from SRC willow and poplar, primarily grown on poor quality marginal land. Similarly, Lovett *et al*. (2009) found that growing *Miscanthus* on low‐grade agricultural land in England would allow for increased planting on approximately 0.35 Mha which would have a minimum impact on UK food security. There is therefore the potential to increase the production of 2G biomass crops without impacting significantly on food crop production (Alexander *et al*., 2014; Hastings *et al*., 2014; Wang *et al*., 2014).

ES include provisioning, regulating, supporting and cultural services, which provide a number of vital services for society and so should be incorporated into decisions related to land use change (Metzger *et al*., 2006). As an exemplar, land use change to 2G feedstock production and impacts on GHG balance and carbon sequestration, can be viewed as a mechanism that will influence the provision of a key ES, namely climate regulation. As such, studies examining this aspect of feedstock production contribute to a growing literature that aims to inform policy by incorporating the value (both monetary and nonmonetary) of ES into the decision‐making process. Publication of the Millennium Ecosystem Assessment (MEA) (Millennium Ecosystem Assessment, 2005) and UK National Ecosystem Assessment (UK National Ecosystem Assessment, 2011), make a compelling case that failure to incorporate such values into land use decision‐making, can result in significant economic and social costs. For example, Bateman *et al*. (2013) demonstrate that incorporating the value of ES into land use planning for the UK could deliver significant benefits for society that are not realized by a focus on agricultural production alone.

Crops such as *Miscanthus* and SRC have also been identified as offering potential positive effects on biodiversity when compared to arable land use (Rowe *et al*., 2009). Biodiversity is a key element of ES (UK National Ecosystem Assessment, 2011), however all of the ecosystem services interact and thus are all important. Processes underpinning ES may also be enhanced under 2G crops including decomposition and predation, but it is difficult to make generalizations given the paucity of data in this area (Rowe *et al*., 2013).

Our ability to ask questions relating to the deployment of 2G crops across the UK has increased substantially over recent years with the development of a number of process based models that enable us to examine different deployment strategies. For example ForestGrowth‐SRC (Tallis *et al*., 2013), MiscanFor (Hastings *et al*., 2009a) and ESC‐CARBINE (Thompson & Matthews, 1989; Pyatt *et al*., 2001) have been developed to model the yield of SRC (willow and poplar), *Miscanthus* and SRF respectively. Models such as these provide valuable insight into potential biomass yield and how this may vary spatially and temporally across the UK, as the climate changes. However, to date they have not considered environmental factors beyond assessing yield supply from different agricultural land classes (Lovett *et al*., 2009; Aylott *et al*., 2010) and the impacts on GHG balance (Hastings *et al*., 2008, 2009b; Dondini *et al*., 2009; Hillier *et al*., 2009; Zatta *et al*., 2014). Here we extend this analysis to provide the first assessment of the likely impact of 2G bioenergy crop transitions on a wide range of ES in temperate environments. We focus on three candidate feedstocks for the UK namely *Miscanthus*, poplar and willow as short‐rotation coppice (SRC) and poplar as short‐rotation forestry (SRF), and transitions from arable land, grassland and forest.

## Materials and methods

**Figure 1:**
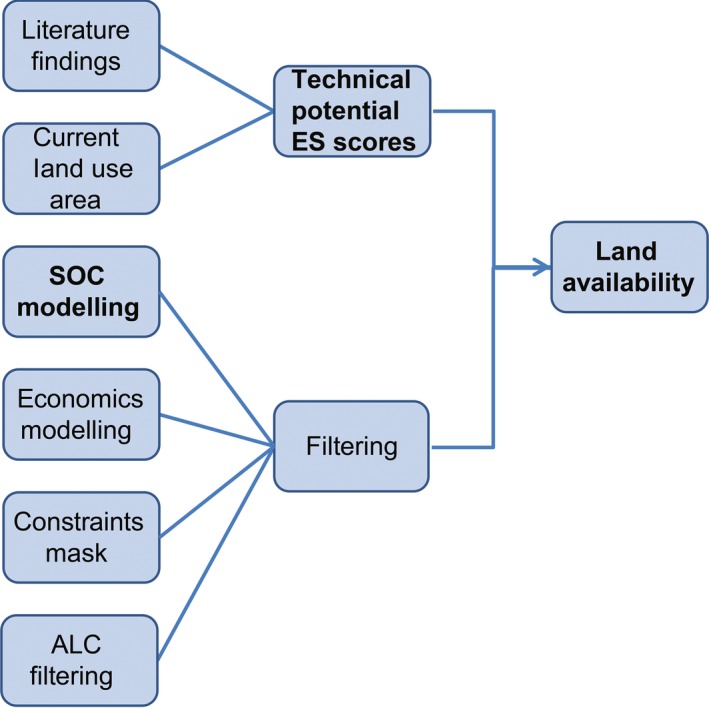
Summarizing schematic of the process of methods involved in producing the estimations of appropriate and available land use transitions and their spatial distributions. Items in bold represent points of output.

The methods used here include a literature‐based search, production of a spatial map of ES effects, SOC change modelling and filtering for suitable land, as summarized in Fig. 1. The different aspects were combined to produce an estimation of the effects of 2G crop production on the land and associated ES where their growth is a viable option.

### Literature‐based search and evaluation of transition effects

Based on a search of ISI Web of Science using the terms ‘biofuel’, ‘biodiesel’, ‘bioethanol’, and ‘bioenergy’ together with keywords relating to commonly examined ES [see Supplementary information Table S1 and Holland *et al*. (2015)], studies were identified that examined land use transitions for three reference states: 1st generation arable crops, grassland and forest (both plantation and natural). For the grassland category, studies that were relevant for transitions from semi‐improved and improved grasslands not used for crop production were selected. References returned by the search were initially filtered for relevance based on their title and abstract. To provide focus and relevance, the UK was used as an exemplar and thus literature examining crops suitable for the UK temperate climate, namely SRC willow and poplar, SRF, and *Miscanthus* were utilized. As some characteristics that may confer ecosystem service benefits (e.g. persistent ground cover) are common across different types of 2G feedstock beyond those that will likely be deployed in the UK we retained studies that detailed other transitions of likely relevance. These were dominated by studies of conversion of arable land to energy grasses in the USA (see Table S2).

The full text of those studies that appeared relevant was obtained and assessed in detail and data on the ES examined, the specific feedstock, the geographical location, the land use transition and whether the study used empirical data collected in the field or was based on a modelling approach (Table S2) was extracted. Transitions were scored as having a positive, negative or neutral effect on an ES based on the statistical analysis presented in the study and the stated results and conclusions of the authors. Studies were selected that measured a direct transition through time from the reference, or used a space for time substitution that contrasted provision of services under a reference state against provision under 2G feedstock production. See Supporting Information (Appendix S1, Tables S1 and S2 and Fig. S1) and (Holland *et al*., 2015) for a full description of this process.

Results from this literature search were combined with other relevant information (see Supporting Information – Appendix S1, Tables S1 and S2 and Fig. S1) to develop a ‘threat matrix’ for ES impacts following transitions to SRC, *Miscanthus* or SRF. The threat matrix was assembled as a summary of all of the analysed literature and confidence assigned based on the amount of information available and agreement between studies. For example the impacts of transitions from arable to *Miscanthus* on Hazard regulation was scored as positive and high confidence as: (i) of 11 studies that considered transitions from arable to second‐generation energy grasses 10 report a positive effect; (ii) a number reviews (Börjesson, 1999; Donnelly *et al*., 2011) and studies (Updegraff *et al*., 2004; Boardman & Poesen, 2006; Lattimore *et al*., 2009; Busch, 2012) explicitly consider how changes in agricultural practice under this transition promotes a reduction in surface runoff (Blanco‐Canqui, 2010) and wind erosion (Busch, 2012; Holland *et al*., 2015). For the same service we found no studies that considered the implication of land use transitions from Forestry/Woodland to *Miscanthus*. As across studies the length of the management cycle emerges as key to understanding the implications of transitions to 2G feedstock production (Lattimore *et al*., 2009; Donnelly *et al*., 2011; Schulze *et al*., 2012) it was considered that this transition would have a negative impact on the provision of this service however, in the absence of specific reference state studies, we assigned low confidence to this. Full discussion of the development of this matrix is provided by Holland *et al*. (2015).

The scoring was designed to reflect the difference in confidence of effects, and it was weighted to reflect this and increase the differences between possible scores out of a potential score of ±126. Fourteen key provisioning and regulating services affected by 2G crops were assessed to develop an ES score. Positive, neutral and negative impacts were scored alongside confidence in the available literature (Table 1).

**Table 1:**
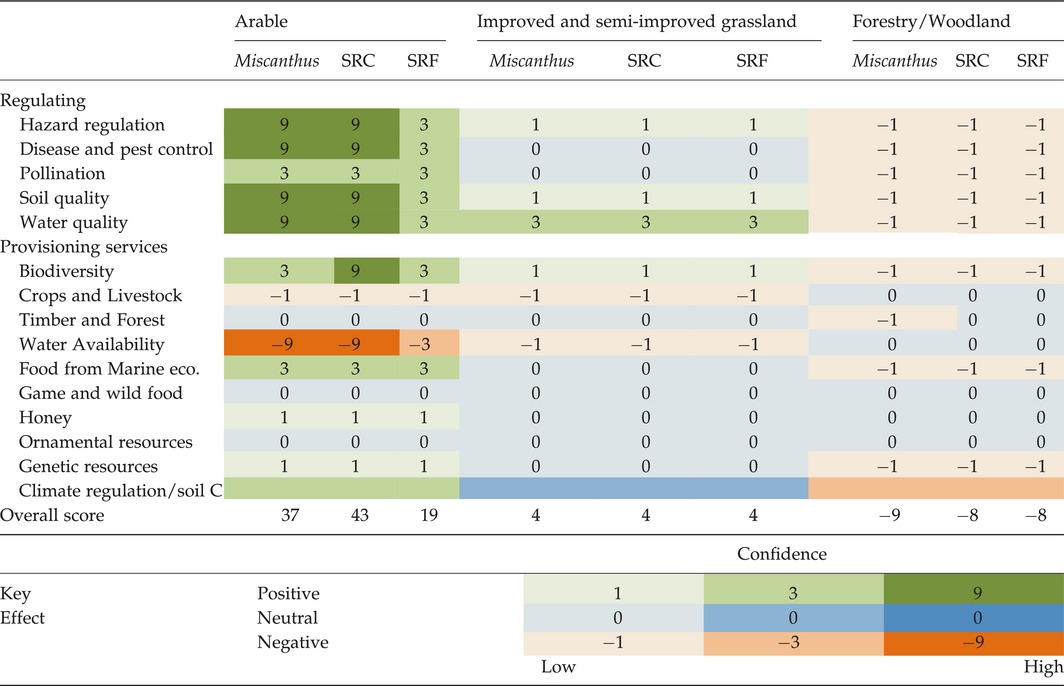
Threat matrix of ecosystem service effects of transitions to differing bioenergy crops

### SOC modelling

An exception to the methods described above was made in the case of climate regulation and soil C; this was because much more quantitative data are available through GHG (Barnett, 2010; Plevin *et al*., 2010; Yan *et al*., 2010) and SOC research (Zimmermann *et al*., 2012; Albaladejo *et al*., 2013) and with modelling able to predict soil C changes for the specific transition of interest. This ES effect category was added to Table 1 using output from the Bossata and Agren cohort soil carbon model (Bosatta & Agren, 1991) incorporated in the MiscanFor model (Hastings *et al*., 2009a). As this category of the threat matrix is model‐derived, it was not included in the ES effect score to produce spatial maps. The model is based on previous land use and SOC content with organic matter input from 2G crop. The model was compared by Dondini *et al*. (2009) to RothC (Coleman & Jenkinson, 1999) for *Miscanthus* crops. The model was run for the mean soil organic carbon (SOC) change (Mg C ha^−1^) per year per cycle of 15 years (standard replanting frequency) for four cycles; 60 years total. This was achieved using *Miscanthus* yields for 2010, the Harmonized World Soils Database (HWSD) soil SOC data (FAO/Iiasa/ISRIC/Isscas/JRC, 2009) and land use data, considering previous land use: forests, arable lands, improved grasslands and all grasslands. All data were at 1 km^2^ resolution.

*Miscanthus* and SRC have similar management in that land disturbance by tillage only occurs in the establishment year after which the only intervention is harvesting and possibly the use of herbicide to control weeds. In addition both *Miscanthus* and SRC have annual leaf fall and root turnover which is approximately one third of the annualized dry matter yield. As the soil carbon is a balance between the decay of the initial soil carbon and the rate of input, and its decomposition rate and the yields for the two 2G crops were similar, *Miscanthus* was taken as a proxy for SOC change under SRC for this analysis. Due to a lack of published experimental data, SOC change was not modelled for SRF. For each 1 km^2^ grid cell the 2G crop with the greatest yield was taken to be the optimum and the SOC change from the cohort model applied to the respective crop.

### Land availability filtering

The land available for planting was calculated using constraints maps produced by Lovett *et al*. (2014) using social and environmental constraints based on 8 factors: road, river and urban areas; slope > 15%; monuments; designated areas; existing protected woodlands; high organic carbon soils; and areas with a high ‘naturalness score’ such as National Parks and Areas of Outstanding Natural Beauty. This land availability was further constrained using agricultural land classes (ALC) (Lovett *et al*., 2014) in GB as summarized in Table 7, accomplished by aggregating a map of the ALC data at 100 m^2^ raster resolution to derive total hectares of land in different ALC in each 1 km^2^ grid cell. The land availability was compared to distributions of planting scenarios at a 1 km^2^ resolution to determine the suitability of planting preferentially on ALC4 then secondarily on ALC3 based on baseline and 2020 planting scenarios reported by Lovett *et al*. (2014) and Alexander *et al*. (2014). As planting scenarios are not available for SRF, only *Miscanthus* and SRC filtered data are presented. Due to the long term investment required for SRF crops, these are used commercially less than *Miscanthus* and SRC. The difference in management strategies also has resulted in fewer research projects on SRF which is a contributing factor to the lack of planting scenarios for SRF crops. Finally these ALC filters were further categorized to assess the proportions of positive ES scores. This was performed to find all areas with positive (ES score ≥0), moderately positive (ES score ≥20) and highly positive (ES score ≥30) ES effects to represent a range of recommendations in order to produce a summary of the ES effects and viable regions in which 2G crops could be planted (Fig. 6).

The SOC change predictions were aggregated to 1 km^2^ grid cells and compared with baseline and 2020 planting scenario data for *Miscanthus* and SRC (Table 2) (Alexander *et al*., 2014; Lovett *et al*., 2014). The planting scenarios were based on mean climate data from 1960–1990 (baseline) or predicted climate data for 2020 from the UKCP09 dataset prepared by the UK Met Office Hadley Centre (Jenkins *et al*., 2009; Hastings *et al*., 2014). These scenarios also used conservative prices of £60 odt^−1^ and £48 odt^−1^ for *Miscanthus* and SRC respectively as current market prices (Alexander *et al*., 2014). The 2020 scenario was based on higher emissions assumptions because this was the alternative which gave rise to the largest increase in planting in the analysis conducted by Alexander *et al*. (2014). On a national scale the SOC change in Mg per hectare per year was divided into four categories. The number of 1 km^2^ grid cells in each of these categories was calculated for GB, baseline and 2020 planting (Table 3). The predicted hectares of planting in each 1 km^2^ cell were subsequently multiplied by the SOC estimated for each region of GB (Table 4).

**Table 2:** Overview of planting scenario and constraints filtering for the SOC change predictions

	Baseline	2020
Climate data	Mean climate data 1960–1990	Predicted data from UKCP09 (Jenkins *et al*., 2009)
Economics data	£60 odt^−1^ (*Miscanthus*) and £48 odt^−1^ (SRC) (Alexander *et al*., 2014)	Prices as per Alexander *et al*. (2014)
Constraints	Social and environmental (Lovett *et al*., 2014) constraints and demand constraints (Wang *et al*., 2014)	
SOC Mg ha^−1^ yr^−1^	−70 to −20, >−20 to −5, >−5 to 0 and >0 to 5	
Geographical regions	GB regions as determined in Lovett *et al*. (2014)	

**Table 3:** National SOC change estimates across GB and in regions identified for planting using the economics model (Alexander *et al*., 2014; Lovett *et al*., 2014) under baseline and 2020 planting scenarios. Land areas are given as ha and percentage of the total area

Soil carbon change (SOC) Mg per ha per year	*Miscanthus* All GB	*Miscanthus* Baseline Planted	*Miscanthus* 2020s Planted	SRC All GB	SRC Baseline Planted	SRC 2020s Planted
	ha; (%)	ha; (%)	ha; (%)	ha; (%)	ha; (%)	ha; (%)
‘−70 to −20’	3 669 500; (16.24)	1200; (0.13)	2600; (0.19)	3 664 400; (16.24)	400; (0.16)	500; (0.19)
‘>−20 to −5’	356 800; (1.58)	800; (0.09)	1300; (0.10)	384 700; (1.70)	600; (0.24)	600; (0.23)
‘>−5 to 0’	2 323 400; (10.28)	2000; (0.22)	2600; (0.19)	2 957 700; (13.11)	3800; (1.50)	4200; (1.63)
‘>0 to 5’	16 242 300; (71.89)	892 300; (99.55)	1 359 500; (99.52)	15 558 200; (68.95)	248 700; (98.11)	253 100; (97.95)
Total	22 592 000; (100)	896 300; (100)	1 366 000; (100)	22 565 000; (100)	253 500; (100)	258 400; (100)

**Table 4:** Predicted SOC change per hectare based on SOC estimates and planting scenarios per region

Geographical region	*Miscanthus*		SRC	
	Base Planted	2020s Planted	Base Planted	2020s Planted
	SOC Chg Mg ha^−1^ yr^−1^	SOC Chg Mg ha^−1^ yr^−1^	SOC Chg Mg ha^−1^ yr^−1^	SOC Chg Mg ha^−1^ yr^−1^
Highlands and Islands			0.85	
North‐eastern Scotland				
Eastern Scotland			1.73	
South‐western Scotland	1.91	1.91	2.03	
North‐east			1.46	1.43
North‐west	1.70	1.74	2.18	2.20
Yorkshire and the Humber	2.28	2.21	2.62	2.69
East Midlands	2.33	2.17	1.00	1.13
West Midlands	2.08	1.66	1.98	1.28
East of England	2.32	2.24		
London				
South‐east	2.76	2.72		1.50
South‐west	2.48	2.48	2.10	1.59
Wales North	1.77	1.56	2.14	2.15
Wales East	1.86	1.78	1.30	1.06
Wales West	2.10	2.09	1.56	1.24
Wales South	2.56	2.49	2.30	2.30
Total	2.28	2.02	2.17	1.96

### ES scores and spatial mapping

In order to gain spatial understanding of how land use transition to bioenergy crops might impact ES across the UK, ES scores were mapped based on different land use constraint scenarios (see Land availability filtering section for details) with the aid of the threat matrix. Spatial analysis was carried out using ArcMap 10.1 (ESRI, Redlands, CA, USA). Firstly, Land Cover Map 2007 categories woodland/forestry (LCM2007 1 and 2), arable (LCM2007 3), grassland (LCM2007 4–8) and ‘other’ (all other LCM2007 categories) were mapped at a 100 m resolution raster. The land use constraint scenarios were subsequently applied to the land cover as follows:

- All available land within our 100 m outline grid
- All available land after applying the constraints mask (see filtering section for details)
- As scenario B but limited to ALC 3–5 (i.e. avoiding the best quality agricultural land)
- As scenario B but limited to ALC 4–5

This data were utilized to summarize the land availability per region with regions determined as in Lovett *et al*. (2014). Also included are total land per region, available hectares of arable, grassland and woodland in each scenario A–D above, and scenario D as a percentage of the total available.

Technical potential ES scores were calculated using the ES effect scores in the threat matrix applied to the land cover distributions. These calculations were based on the percentages of each crop present for each 1 km^2^ grid cell of GB. For this, the sum of each ES effect score was multiplied by the respective percentage of each land cover for each 1 km^2^ grid cell for each given land use transition scenario:

$$\begin{array}{c} {\text{The technical potential ES score per km}}^{2}=(\%\text{arable land cover}\times \text{ES score of transition of arable}\;\text{to chosen crop})+\left(\%\text{improved grassland cover}\times \text{ES score of transition of grassland to chosen crop}\right)+\left(\%\text{woodland cover}\times \text{ES score of transition of woodland}\;\text{to chosen crop}\right) \end{array}$$

For the spatial mapping of the ES scores, improved grassland cover was utilized to best represent grassland category (improved and semi‐improved grassland) in the threat matrix as literature used often did not distinguish between the categories. This is despite the Land Cover Map 2007 distinguishing improved grassland from natural/neutral and seminatural/semineutral grasslands through higher productivity, lack of winter senescence and location and/or context.

The predicted ES effects were summarized per region in each of the LCM2007 scenarios described above. This gave the average ES score per region for available land in each scenario/crop combination.

## Results

### Literature‐based search and production of ES scores

The effect of each bioenergy land use transition on ES is predominantly governed by the initial land uses (Table 1) and, to a lesser extent, linked to the underpinning research available for a particular crop type. When changing from improved and semi‐improved grassland, the choice of bioenergy crops had no overall impact on the ES score with each transition giving an ES score of 4. These transitions were largely governed by neutral effects on ES suggested by the available literature. In general, loss of forestry/woodland had a negative impact on ES score, irrespective of bioenergy crop type (Table 1). Choice of bioenergy crop had only a small effect on transitions from forestry/woodland, with the two short‐rotation woody crops (SRC and SRF) and *Miscanthus* scoring −8 and −9, respectively. Bioenergy crop choice had a more pronounced and positive effect for the transition from arable land use, with *Miscanthus*, SRC and SRF scoring 37, 43 and 19 respectively, reflecting a well‐developed understanding of the implications of different transitions and considerable published research evidence to confirm this metric. As considerably fewer papers are available in the literature on the ES effects of transitions to SRF, the confidence level was scored lower, creating a lower overall ES impacts score and thus impacting on results.

### Soil organic carbon change

Detailed analysis of soil C shows for the vast majority of 2G crop planting, and a net increase in SOC is likely, especially after constraints are applied. Blanket planting of *Miscanthus* or SRC across GB would result in 71.89% and 68.95% of planted land, respectively, with increased SOC ha^−1^ yr^−1^ (Table 3). When restricted to baseline planting scenarios identified by the economics model (Alexander *et al*., 2014) and constraints mask (Lovett *et al*., 2014), 99.55% of land transitioned to *Miscanthus* was predicted to result in a positive SOC change. In the same planting scenario with a transition to SRC, 98.11% land was identified to result in a positive SOC change. In the 2020 planting scenarios, these were similarly 99.52% and 97.95% of land, respectively. This contrasts with the percentage of land for which a negative impact on SOC (a net carbon release) was predicted. Only 0.13% land in a transition to *Miscanthus* was recorded as resulting in net CO_2_ emissions using the baseline planting scenario. For SRC, this was only marginally more at 0.16% of land area. With 2020 planting scenarios, this predicted land area was 0.19% of land area for both *Miscanthus* and SRC. In each planting scenario, this equates to a maximum of 2600 ha land, and these areas with a predicted carbon emission generally corresponded to areas with a high initial SOC.

The regional analysis of SOC (Table 4 and supplementary Table S3) showed that no overall negative SOC changes were found. Generally regional impacts ranged from 1.5 to 2.5 Mg C ha^−1^ yr^−1^ net gains in soil carbon for the first 15 year cropping cycle, in transition from current land uses outside the constrained areas (Lovett *et al*., 2014) to *Miscanthus* or SRC. Ranking the SOC per region per 2G crop suggests that for both planting scenarios, south‐east, south‐west and South Wales have the highest SOC for *Miscanthus*, whereas north‐west, Yorkshire and the Humber and South Wales have the highest SOC for SRC.

**Figure 2:**
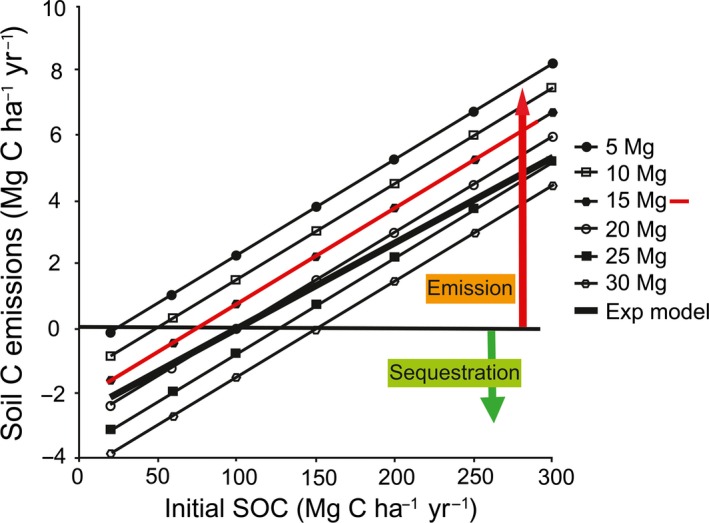
The modelled relationship between soil C emissions and initial SOC within the top 30 cm of soil when planting *Miscanthus*. The red sloping line (15 Mg) represents the mean peak surface biomass for the Midlands, UK harvest yield of 10 Mg ha^−1^.

Figure 2 illustrates the relationship between above‐ground biomass yield to initial SOC. The red line (15 Mg C ha^−1^ yr^−1^ dry matter) represents the mean peak surface biomass (typical for the Midlands, UK), which gives a harvested biomass of 10 Mg C ha^−1^ yr^−1^ dry matter (Fig. 2). The model shows that equilibrium SOC for *Miscanthus* is around 100 Mg C ha^−1^ in the top 30 cm, so that a soil with SOC below 100 Mg C ha^−1^ will gain C, whereas above 100 Mg C ha^−1^ will lose C.

**Figure 3:**
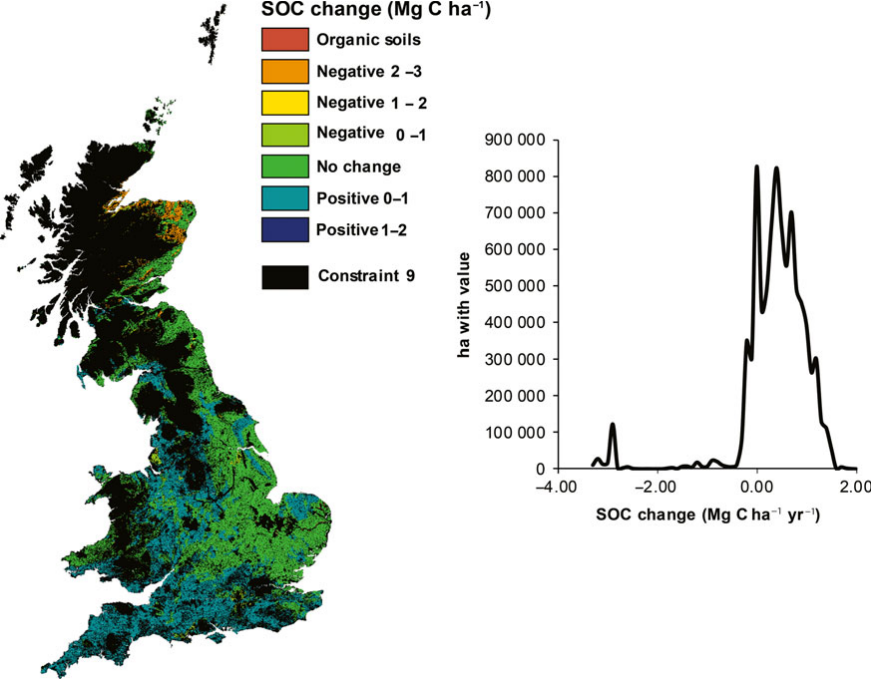
The spatial distribution of technical potential of SOC change for the United Kingdom when planting *Miscanthus* on arable land. SOC change found using the MiscanFor model with a 1 km^2^ resolution. Constraint 9 is based on eight factors used by (Lovett *et al*., 2014) such as slope, monuments, existing woodlands and areas with high ‘naturalness score’.

**Figure 4:**
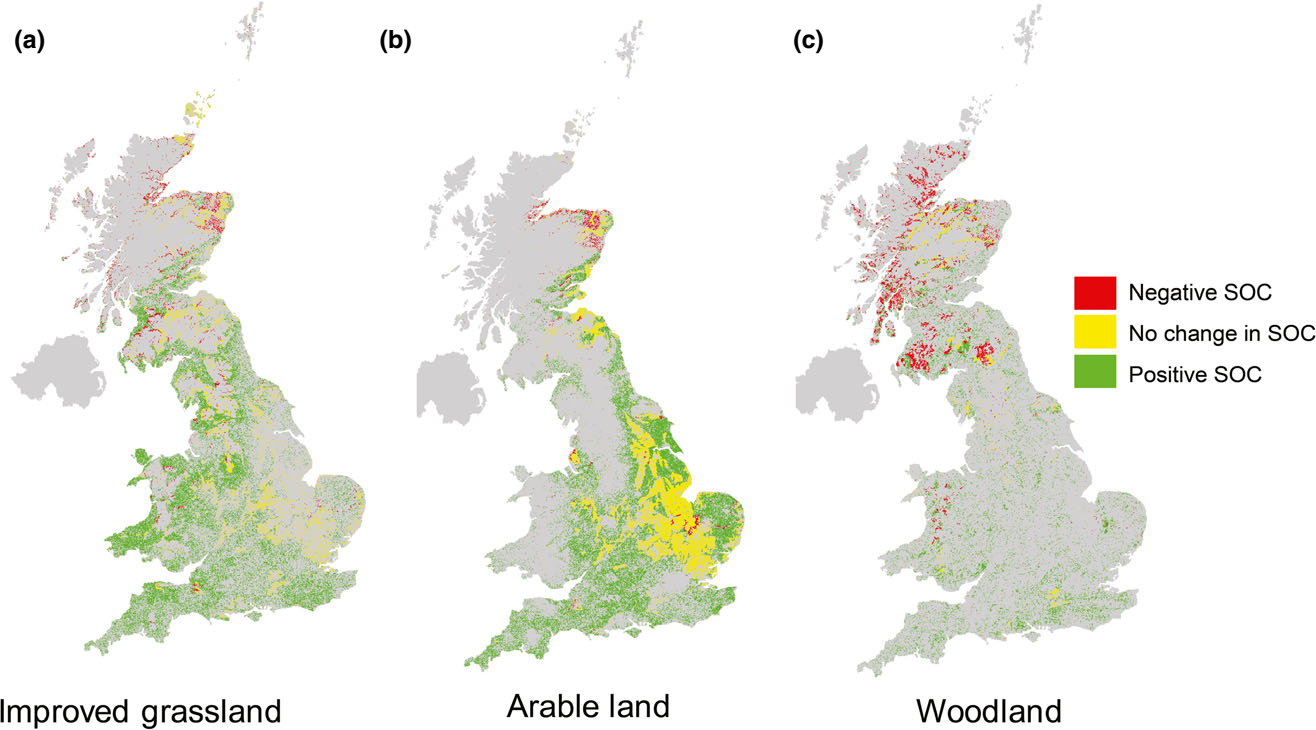
The predicted spatial distribution of SOC change when planting *Miscanthus* in the United Kingdom for previous land use categories of improved grassland (a), arable land (b) and woodland (c).

Fig. 3 confirmed either no change or a gain of SOC (positive) through planting *Miscanthus* on arable land across England and Wales and only a loss of SOC (negative) in parts of Scotland. The total annual SOC change across GB in the transition from arable to *Miscanthus* if all nonconstrained land was planted with would be 3.3 Tg C yr^−1^. The mean changes for SOC for the different land uses were all positive when histosols were excluded, with improved grasslands yielding the highest Mg C ha^−1^ yr^−1^ at 1.49, followed by arable lands at 1.28 and forest at 1. Separating this SOC change by original land use (Fig. 4) reveals that there are large regions of improved grasslands which, if planted with bioenergy crops, are predicted to result in an increase in SOC. A similar result was found when considering the transition from arable land; however for central eastern England, there was a predicted neutral effect on SOC. Scotland, however, is predicted to have a decrease for all land uses, particularly for woodland due mainly to higher SOC and lower *Miscanthus* yields and hence less input.

### Land availability filtering and spatial ES impacts

**Figure 5:**
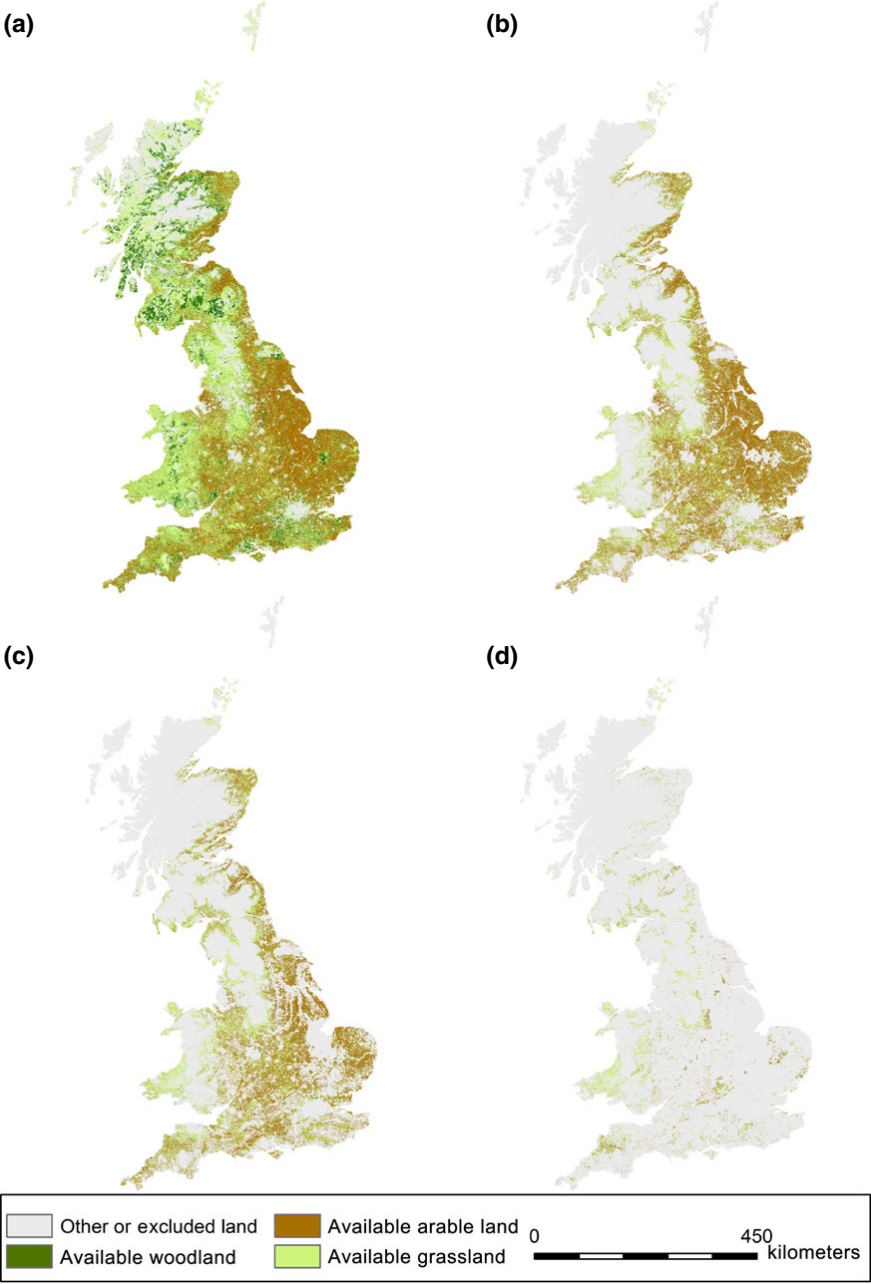
The spatial distribution of current land use and the availability of land for LUC transitions. Land use categories include arable (LCM07 3), woodland (LCM07 1 and 2), grassland (LCM07 4–8) and other (all other crop types and excluded regions). (a) All available land within the 100 m outline grid, (b) all available land also within the UKERC9 constraint mask, (c) as with b but also on ALC 3–5, (d) as with b but also on ALC4–5.

To assess overall spatial changes in ecosystem service (ES) impacts using transitions summarized in Table 1, only current land use data of woodland, improved grassland and arable land were assessed. Of these land use types, Fig. 5 shows the current land cover crop which will subsequently determine the ES score for transition to bioenergy cropping. It also shows that when filtering the land availability by the constraints mask and ALCs, as detailed in the Methods section, the land available for transitions to 2G crops is limited particularly in Scotland, Wales and NW England. In general, in Scotland and mid‐Wales – the most widely planted land used was woodland, in the east of England, it was arable, and in the west of England and Wales, it was improved grassland. Consequently, the largest positive benefits of LUC to 2G crops for ES are predicted to occur in the east of England as the transition from arable has the greatest impact on ES scores, at least partially because such transitions have high confidence score following several empirical studies reported in the literature (Table 1).

An assessment of available land for 2G crop transitions in each scenario (Table 5) revealed Wales and Scotland to have the highest percentage of suitable land in ALC 4 and 5, with land classified into ALC 3 and 4 more evenly distributed across GB. When ES impacts were included in the regional assessments, transition to SRC had the most positive ES impact, followed by *Miscanthus* (Table 6). For each transition, the five regions with the highest ES score are the east of England, followed by East Midlands, Yorkshire and the Humber and West Midlands. When restricting to the ALC 3–5 or ALC 4–5, the south‐east and south‐west are in the top five with the highest ES scores combined with land available, suggesting these are regions of significant interest.

**Table 5:** Regional land availability of arable, grassland and woodland in each LCM07 scenario

Region Name	Total Hectares	Available Hectares of Arable, Grassland + Woodland in each Scenario				LCM07D as %
		LCM07A ha	LCM07B ha	LCM07C ha	LCM07D ha	
Highlands and Islands	3 933 796	1 760 442	122 859	110 380	55 942	1.4
North‐eastern Scotland	733 111	544 622	299 868	286 782	34 908	4.8
Eastern Scotland	1 812 941	1 293 354	441 609	337 979	88 461	4.9
South‐western Scotland	1 306 783	1 030 373	217 998	210 751	126 244	9.7
North‐east	858 556	637 455	324 597	296 466	57 720	6.7
North‐west	1 413 195	1 047 318	437 998	354 333	67 500	4.8
Yorkshire and the Humber	1 541 067	1 220 499	749 701	472 794	72 229	4.7
East Midlands	1 562 615	1 406 193	1 043 873	736 961	61 809	4.0
West Midlands	1 300 316	1 149 686	760 650	567 200	83 437	6.4
East of England	1 909 478	1 732 398	1 277 537	733 505	62 244	3.3
London	157 397	48 860	18 568	10 606	448	0.3
South‐east	1 907 874	1 662 926	925 504	713 433	148 948	7.8
South‐west	2 382 600	2 186 761	1 114 249	961 032	194 299	8.2
Wales North	617 035	500 925	150 838	133 273	64 983	10.5
Wales East	519 611	463 956	94 860	93 534	82 673	15.9
Wales West	576 851	542 225	205 472	201 031	141 572	24.5
Wales South	363 000	290 082	86 653	69 003	32 552	9.0
Total	22 896 226	17 518 075	8 272 834	6 289 063	1 375 969	6.0

**Table 6:** Regional ES effect per hectare for each LCM07 scenario with transitions to *Miscanthus*, SRC or SRF

Biomass crop	*Miscanthus*				SRC				SRF			
Scenario	LCM07A	LCM07B	LCM07C	LCM07D	LCM07A	LCM07B	LCM07C	LCM07D	LCM07A	LCM07B	LCM07C	LCM07D
Region Name	ES/ha				ES/ha				ES/ha			
Highlands and Islands	0.9	11.5	10.8	6.7	1.4	13.1	12.3	7.4	0.7	7.0	6.7	4.8
North‐eastern Scotland	12.3	20.6	20.3	12.3	14.6	23.8	23.5	14.3	6.4	11.2	11.1	6.9
Eastern Scotland	10.6	23.8	21.8	12.7	12.6	27.6	25.3	14.8	5.7	12.6	11.7	7.2
South‐western Scotland	3.2	11.3	11.4	9.6	4.0	12.9	13.0	11.0	1.8	6.8	6.9	6.0
North‐east	12.2	20.5	20.1	10.1	14.2	23.7	23.2	11.4	6.7	11.2	11.0	6.3
North‐west	9.4	15.9	14.2	10.1	10.8	18.3	16.2	11.4	5.7	9.1	8.3	6.4
Yorkshire and the Humber	20.4	28.2	25.9	15.4	23.6	32.7	30.0	17.7	10.9	14.8	13.7	8.9
East Midlands	25.0	29.1	27.6	18.4	29.0	33.8	32.0	21.3	13.2	15.2	14.5	10.2
West Midlands	18.1	22.6	21.2	16.7	21.0	26.1	24.5	19.1	9.9	12.2	11.6	9.5
East of England	25.8	29.3	28.1	23.8	30.1	34.1	32.7	27.7	13.4	15.2	14.7	12.4
London	6.9	13.9	15.5	9.4	8.2	16.1	17.9	10.9	4.1	7.8	8.5	5.6
South‐east	15.5	24.1	23.7	19.6	18.2	28.0	27.5	22.6	8.2	12.8	12.6	10.7
South‐west	16.0	23.0	22.6	18.2	18.6	26.6	26.1	21.0	8.7	12.4	12.2	10.1
Wales North	5.4	10.5	9.6	7.6	6.1	11.9	10.8	8.4	3.7	6.7	6.3	5.3
Wales East	5.1	10.6	10.6	9.9	5.8	12.0	12.0	11.1	3.7	6.8	6.8	6.5
Wales West	4.6	7.6	7.5	6.3	5.2	8.4	8.3	6.9	3.5	5.4	5.3	4.8
Wales South	6.7	14.1	14.2	11.5	7.9	16.2	16.3	13.2	4.0	8.1	8.2	6.8
Total	13.7	23.4	21.7	13.5	15.9	27.1	25.1	15.5	7.4	12.6	11.8	7.9

**Figure 6:**
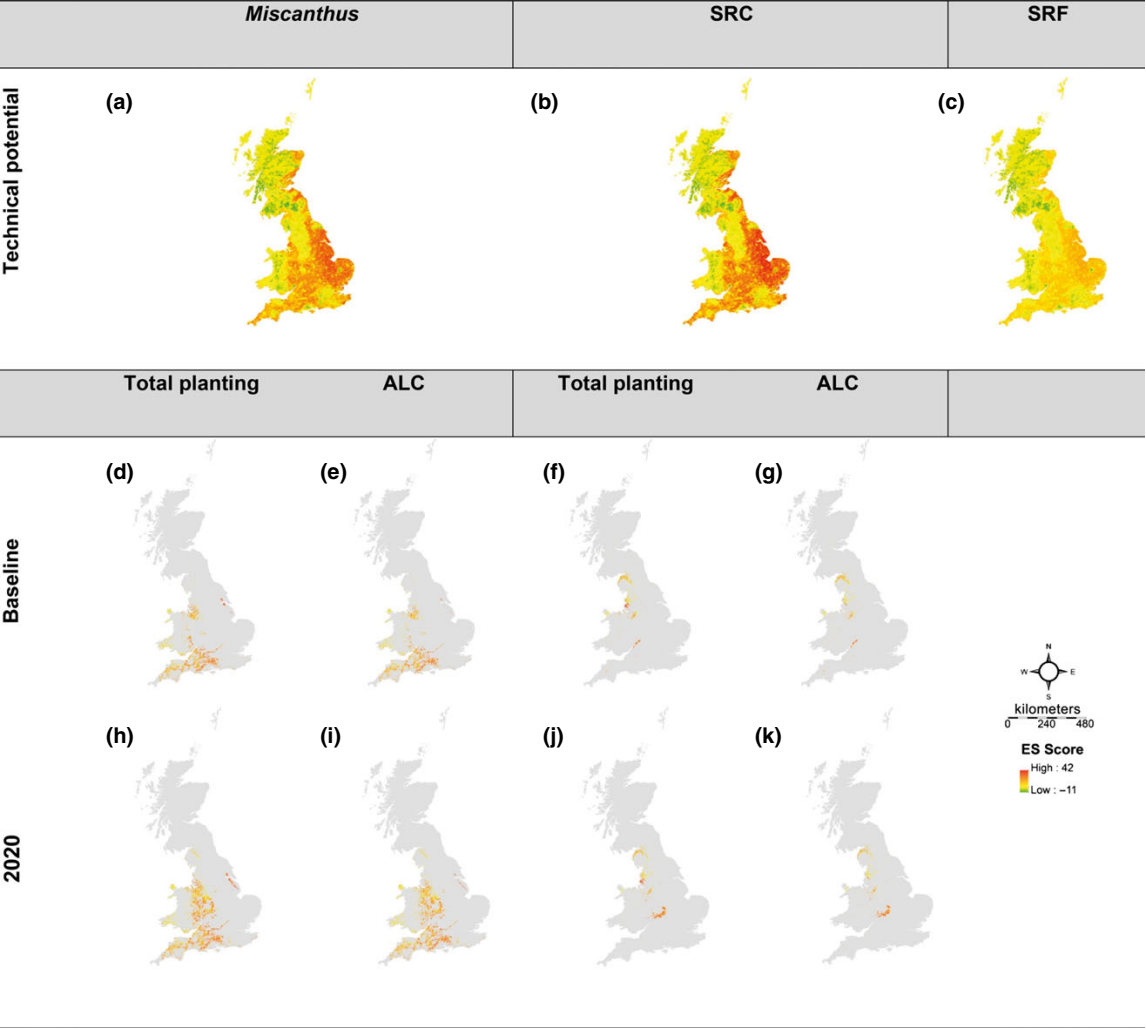
The predicted spatial distribution of technical potential ES effect in GB when planting *Miscanthus*, SRC and SRF (a–c respectively) and the ES effects when restricting planting of *Miscanthus* and SRC to the constrained baseline and 2020 planting scenarios (d–k).

A detailed assessment of potential ES scores was made based on the individual percentage cover for the United Kingdom of the three current land use types in transition to the three bioenergy crops, producing the technical potential ES effect of these transition scenarios (Fig. 6a–c). A minimal difference was observed between transitions to *Miscanthus* and SRC which exceeded the benefits of transitioning to SRF, although transition to SRC indicated a larger positive effect than *Miscanthus* in east England due to biodiversity. For all three energy crop transitions, the smallest benefit of land transitions for ES score was seen in regions where woodland and semi‐improved grassland dominate the landscape (Fig. 5). Although these ES effects are based on percentage cover of the three current land use types transitioning to the three different energy crops at a 1 km^2^ resolution, it is only regions where arable crops dominate that the effect of specific choice of 2G crops is relevant.

Although the technical potential ES effect is informative, the land availability combining the economics model and the baseline and 2020 planting scenarios are crucial for understanding and thus were calculated and filtered for ALC 3 and 4 (Table 7). For the baseline scenario with the economics filter, there was an estimated 169 171 ha that are economically viable for *Miscanthus* planted in GB, of which 40 517 ha can be allocated to ALC 4. After ALC 4 is planted, a remaining 106 575 ha could be planted on ALC 3 land leaving 22 079 ha (13.05%) unallocated to ALC 3 and 4. The baseline planting of SRC, however, requires 88 407 ha in GB of which 16 546 ha can be allocated to ALC 4 and a further 55 959 ha to ALC 3, leaving 15 902 ha (17.99%) unallocated to ALC 3 and 4. This is in contrast to the 2020 planting scenario where for *Miscanthus,* there is 350 263 ha that are economically viable within GB, of which 74 017 ha can be allocated to ALC 4 and 220 295 ha can be allocated to ALC 3 leaving 55 951 ha (15.975%) unallocated. For SRC, this is a similar story where 112 870 ha is predicted to be economically viable in the 2020 planting scenario, of which 18 137 ha can be allocated to ALC 4 and 73 927 ha can be allocated to ALC 3 leaving 20 806 ha (18.43%) unallocated. With ALC 3 and 4, the land available which offers the most positive ES effect is in south‐west England for *Miscanthus* and west and central England for SRC (Fig. 6d–k).

**Table 7:** Land availability and predicted ES impacts of planting of *Miscanthus* and SRC in different ALC for GB after filtering for planting scenarios (Lovett *et al*., 2014). For the baseline scenario much of the unallocated SRC planting is in Lancashire on Grade 1 or 2 land

	ES score	Baseline *Miscanthus* Hectares; (% of planting)	Ha per ES score	Baseline SRC Hectares; (% of planting)	Ha per ES score	2020 *Miscanthus* Hectares; (% of planting)	Ha per ES score	2020 SRC Hectares; (% of planting)	Ha per ES score
Total Planting	≥0	169 171	168 508	88 407	87 691	350 263	348 805	112 870	112 087
	≥20		69 020		19 858		133 101		39 923
	≥30		20 345		7469		36 670		18 307
Allocatable to Grade 4	≥0	40 517; (23.95)	40 141	16 546; (18.72)	16 188	74 017; (21.13)	73 302	18 137; (16.07)	17 712
	≥20		6567		947		10 176		2181
	≥30		599		98		1154		551
Unallocated on Grade 4		128 654; (76.05)		71 861; (81.28)		276 246; (78.87)		94 733; (83.93)	
Remainder Allocated to Grade 3	≥0	106 575; (63.00)	106 442	55 959; (63.30)	55 702	220 295; (62.89)	219 945	73 927; (65.50)	73 606
	≥20		49 879		10 667		90 553		25 356
	≥30		15 077		2021		23 652		10 015
Unallocated on Grades 3 and 4	≥0	22 079; (13.05)	21 925	15 902; (17.99)	15 801	55 951; (15.97)	55 558	20 806; (18.43)	20 769
	≥20		12 574		8244		32 372		12 386
	≥30		4669		5350		11 864		7741

To identify key areas of interest, it would be informative to combine the planting scenarios (baseline and 2020) with land suitability information (both economically and physically) and also predicted ES effect. Therefore, the filtered land availability was assessed for potential ES effect. Of the total planting area available in each planting scenario (Table 7), the percentage of predicted positive ES scores were calculated to be 99.61% for *Miscanthus* baseline, 99.19% for SRC baseline, 99.58% for *Miscanthus* 2020 and 99.31% for SRC 2020. This implies that planting 2G crops in the identified regions would be economically viable and have a positive GHG benefit and an overall positive ES effect. Of the land with a predicted positive ES effect, it is not viable to plant 13.01% (*Miscanthus* baseline), 18.02% (SRC baseline), 15.93% (*Miscanthus* 2020) and 18.53% (SRC 2020) in ALC 3 and 4. This suggests that of the land available to plant 2G crops on, a high proportion would offer a positive ES effect.

The spatial mapping of all land (the maximum technical potential representing the achievable ES scores if LUC was to occur) detailed in Table 7 (Fig. 6a–c), and filtering of the technical potential ES effect (Fig. 6d–k) indicates south‐west England is a key region to target for *Miscanthus* in both baseline and 2020 planting scenarios. In 2020, additionally, this area increases northwards into the West Midlands. When planting SRC, key regions indicated are the north‐west England and parts of East and West Midlands. Due to the rigorous constraints masks (Lovett *et al*., 2014), these regions have the most informed recommendation for planting 2G crops based on economic modelling, SOC modelling and literature‐based assessments of ES.

## Discussion

This study has provided the first assessment of the potential impact of land use transitions to 2G bioenergy crops on the delivery of ES and resolved spatially for GB. The study identified significant differences in potential to deliver positive ecosystem benefits, depending on transition, geographical area, land quality and bioenergy crop type. The approach to evaluating ES suggests that the growth of 2G bioenergy crops across GB broadly produces beneficial effects when replacing first‐generation crops (Table 1). Beneficial effects on the overall ecosystem rather than specific ES are in agreement with recent reports in the literature (Semere & Slater, 2007a,b; Rowe *et al*., 2009; Dauber *et al*., 2010). Benefits of a transition to 2G crops include increased farm‐scale biodiversity (Rowe *et al*., 2011), improved functional attributes such as predation (Rowe *et al*., 2013) and a net GHG mitigation benefit (Hillier *et al*., 2009). Benefits are primarily consequence of low inputs and longer management cycles associated with 2G crops (Clifton‐Brown *et al*., 2008; St Clair *et al*., 2008). The benefits may have distinct temporal patterns as establishment and harvest phases of 2G crop production are disruptive and have a short‐term negative impact on ES (Donnelly *et al*., 2011), although practices could be tailored to ameliorate these; however, this temporal effect has not been considered here and is similar to harvesting and planting food crops, grass or trees.

The threat matrix is novel and revealed the effect of land use transitions on ES from grassland was broadly independent of 2G bioenergy crop choice based on our current understanding. The differences in ES score between bioenergy crops were most significant when transitioning from arable land use, due to positive effects on services including biodiversity, water quality and availability, and hazard regulation (Manning *et al*., 2014; Holland *et al*., 2015).

Spatial application of the ES scores outlined in Table 1, applied across the United Kingdom, revealed the greatest technical potential for ES improvement in east England, where arable crops currently dominate. There are also few differences between *Miscanthus* and SRC so, based on ES improvement effects alone, east England has emerged as the best region for planting these crops. However, transitions throughout these areas are not realistic for various reasons, predominantly due to the need to produce food. Also, analysing each transition in isolation is not fully representative as it is more likely that transition will occur to a mixture of 2G crops to limit the impact of factors such as pest and diseases arising from single cropping over large areas, thus enabling growers to spread risk. However, these transitions provide some insight into the potential impacts in given regions. Also, although hypothetical and not spatially synergistic as analysis of each 1 km^2^ region does not account for neighbouring regions, this analysis provides an indication of potential spatial ES effects in the LUC scenarios.

In these identified areas of eastern England, SRC willow and poplar are predicted to have a reduced performance due to a lower annual rainfall compared to the rest of GB (Tallis *et al*., 2013; Hastings *et al*., 2014) and the same reduced yield is predicted for *Miscanthus* (Hastings *et al*., 2014). Thus, a trade‐off between yield and provision of other ES is emerging, which has relevance for policy development for land management and bioenergy strategy. Comparing the two crops in eastern Scotland, in north‐east England, and in Cambridgeshire where arable crops currently dominate suggests poplar will deliver better yields than *Miscanthus*, although in most other regions, *Miscanthus* is favoured over SRC (Hastings *et al*., 2014). Thus, large ranges in yield and ES effects exist over the country, but the governing factor determining the ES effect is previous land use history rather than the region. Therefore, the best ES improvement is likely to be SRC or a mix of SRC and *Miscanthus* planted on ALC 3–5 land.

Studies such as Aylott *et al*. (2010) proposed ALC 4 and 5 to be the land available for bioenergy production with ALC 1–3 reserved mostly for food production. Planting on ALC 4 and 5 land in England and Wales was predicted to have largely neutral or positive ES effects, and there is little difference according to which 2G crop is established. There is, however, ALC 4 and 5 land in Scotland with a predicted negative ES effect (Fig. 6a–c) although planting in this area would involve a trade‐off with yield. SRC willow and poplar are often predicted to have higher yields in Scotland due to the wetter climate (Tallis *et al*., 2013; Hastings *et al*., 2014). Currently, the MiscanFor model predicts lower yields for *Miscanthus* in Scotland compared to England (Wang *et al*., 2012; Hastings *et al*., 2014), so in these areas, due to a similarity in ES effects when changing from woodland, one of our key findings is that it would be beneficial to plant SRC rather than *Miscanthus*. After applying all filtering, the planting of *Miscanthus* is predicted to be beneficial in terms of ES in the south‐west, whereas transitioning to SRC is predicted to be beneficial in north‐west England. These regions were also identified by Hastings *et al*. (2014) for high yield for *Miscanthus*, a medium yield for SRC willow and high yield for SRC poplar and SRF poplar.

The SOC change modelling (Fig. 4) suggests transitions from grassland and arable land will have an overall positive effect on SOC, particularly in south and west GB. A transition from woodland suggests a largely negative SOC, particularly in Scotland. This is through a loss of standing biomass and subsequently a loss of SOC through harvest, although this is dependent on forest age. For transitions to *Miscanthus* or SRC in baseline and future scenarios (Table 4), spatial variability through South Wales suggested a high SOC, indicating more research in this area is needed. In transition to *Miscanthus,* south‐east and south‐west England were positive in current and future scenarios, whereas for SRC, the most positive effect on SOC occurs in Yorkshire and the Humber, north‐west England and North Wales. The SOC change per region of 0.85–2.76 Mg C ha^−1^ yr^−1^ was predicted which is higher than many studies have found for *Miscanthus* (Matthews *et al*., 2001; Clifton‐Brown *et al*., 2007), and although our range overlaps that of Hansen *et al*. (2004), the SOC rate predicted here is still higher. The sequestration rate, however, is similar to the range of 1.07–1.46 Mg C ha^−1^ yr^−1^ found by converting arable land to native vegetation (Smith *et al*., 2008; Zatta *et al*., 2014). Dondini *et al*. (2009) found a high carbon sequestration rate for *Miscanthus* established on arable land, whereas Zatta *et al*. (2014) found little change when established on semipermanent grassland. Differences between crops are attributable to differences in C_3_ (SRC) and C_4_ (*Miscanthus*) plant input rates and differences in their photosynthetic pathways (Wynn & Bird, 2007). Here, we show SRC has the potential to match SOC change of *Miscanthus,* and both crops may have a higher maximum potential than previously thought.

The ability of 2G crops to sequester SOC will largely depend on the previous land use and its resulting initial SOC. This explains the variation in SOC change in the literature for both 2G crops. Comparisons of SOC change between SRC and *Miscanthus* at the same site are rare, but Borzecka‐Walker *et al*. (2008) found that net soil carbon sequestration for *Miscanthus* in their trial was 0.64 Mg C ha^−1^ yr^−1^, whereas for willow, it was 0.30 Mg C ha^−1^ yr^−1^. This indicated the two 2G crops may differ; however, both 2G crops would be more comparable to each other than to first‐generation biofuel crops or agro‐ecosystems (arable or grassland). Comparison of the SOC changes under 2G crops is an area for future research, and as a change back to arable crops will result in an SOC reduction, this should also be considered.

Compiling the threat matrix highlighted significant gaps in our understanding of the implications of land use transitions for many of the ES considered here, consistent with findings of other studies (Donnelly *et al*., 2011). Results were driven by transitions where the evidence base is strongest (see Table 1), but as understanding on ES increases, changes could alter our conclusions. An area for further analysis relates to landscape‐scale effects associated with commercial scale production on the provision of services, as a number of studies suggest there may be significant implications of commercial scale 2G feedstock deployment (Bianchi *et al*., 2006; Vanloocke *et al*., 2010; Bourke *et al*., 2014) not revealed at smaller scales.

A further limitation of the results is that they consider changes in the provision of the service associated with a transition but do not consider the context in which this is occurring or synergies between services. Ultimately, the interplay of environmental factors such as water resource availability and social factors such as societal demand for a particular ecosystem service and/or the adaptive capacity of groups will influence the impact of land use transitions. For example, the higher seasonal water use of 2G crops due to their large root systems, high leaf area index and strong coupling with the atmosphere (Finch & Riche, 2010; Le *et al*., 2011) that can negatively affect water resources may not be relevant where patterns of water availability match periods of crop demand or if there was investment in efficient irrigation procedures.

The complexity of such analysis can be highlighted with an example of the transition of marginal land to 2G crops production. Although Lovett *et al*. (2009) and Aylott *et al*. (2010) highlight relatively large areas of marginal land in the United Kingdom suitable for 2G crops with minimal impact on food production, Kang *et al*. (2013) suggest the importance of marginal land for food and traditional forage–livestock production could be underestimated, leading to direct competition between food and fuel production. More research may help clarify the use of marginal land in the United Kingdom. Further research will also aid the understanding of the relative importance of specific ES which might indicate that a weighted analysis would be more appropriate, although progress in this area requires further landscape‐scale empirical work including manipulative field experiments.

This research has highlighted the complex relationships that exists in managing a multifunctional landscape. There is a need for a balance between protecting ES, meeting food and fuel demands, which may involve permitting a reduced yield. Limited data are available on the impacts of bioenergy cropping on a range of ES beyond that of GHG balance and carbon footprinting. Other studies have quantified impacts on biodiversity (Dauber *et al*., 2010), but this study is one of only a few to consider a wider range of services (Metzger *et al*., 2006; Werling *et al*., 2014), alongside yield potential for a range of land use transitions and 2G crop types. Given the paucity of data for many of the transitions [see Supporting Information (Appendix S1, Tables S1 and S2 and Figure S1)], the results presented in Table 1 represent our current understanding and highlight areas for future work, notably on the implications of transitions from improved and semi‐improved grassland on the provision of ES. As the evidence base improves, it is possible to update the analysis presented here to reflect this new knowledge and further refine our understanding of desirable deployment strategies.

Our analysis offers a preliminary consideration of the available evidence but also highlights a number of key trends relevant to the development of sustainable intensification strategies that optimize ES within a limited land resource, such as that in GB. When land is filtered for different planting scenarios under ALC 3 and 4, >92.3% available land will offer a positive ES effect when planting *Miscanthus* or SRC and such transitions are likely to create a net improvement in GHG balance. Ideally, a regional network of commercial scale plantations, with monocrop and mixtures of SRC and *Miscanthus,* could now be initiated to test our hypotheses on the benefits of these crop types for transitions from arable and grassland, where the full range of ES are quantified in empirical studies at landscape scale, such as that suggested by Manning *et al*. (2014). Research into social factors will also be important for the acceptability of the different crops, and the public value of specific services, particularly those related to amenity, has not been considered in great detail here as this research has been carried out (Upham & Shackley, 2007; Selman, 2010; Dockerty *et al*., 2012).

## Supporting information


**Appendix S1.** Detailed approach taken to compile the ecosystem service impact matrix.
**Figure S1.** Flow chart of steps taken in compiling threat matrix.
**Table S1.** Results of literature review indicating ecosystem services examined, keywords used in the Web of Science search and the total references after each of the filtering criteria were applied.
**Table S2.** Studies that use a reference state approach to examine the implications of transitions to 2G bioenergy feedstocks.

**Table S3.** Predicted land availability and SOC change per region of GB based on SOC estimates and planting scenarios per region.

## References

[gcbb12263-bib-0001] Albaladejo J , Ortiz R , Garcia‐Franco N , Navarro AR , Almagro M , Pintado JG , Martínez‐Mena M (2013) Land use and climate change impacts on soil organic carbon stocks in semi‐arid spain. Journal of Soils and Sediments, 13, 265–277.

[gcbb12263-bib-0002] Alexander P , Moran D , Smith P *et al* (2014) Estimating UK perennial energy crop supply using farm‐scale models with spatially disaggregated data. GCB Bioenergy, 6, 142–155.

[gcbb12263-bib-0003] Arima EY , Richards P , Walker R , Caldas MM (2011) Statistical confirmation of indirect land use change in the Brazilian Amazon. Environmental Research Letters, 6, 024010.

[gcbb12263-bib-0004] Aylott M , Farrall K , Casella E , Taylor G (2010) Estimating the supply of biomass from short‐rotation coppice in England, given social, economic and environmental constraints to land availability. Biofuels, 1, 719–727.

[gcbb12263-bib-0005] Barnett MO (2010) Biofuels and greenhouse gas emissions: Green or red? Environmental science & technology, 44, 5330–5331. 20553045 10.1021/es101309q

[gcbb12263-bib-0006] Bateman IJ , Harwood AR , Mace GM *et al* (2013) Bringing ecosystem services into economic decision‐making: Land use in the United Kingdom. Science, 341, 45–50. 23828934 10.1126/science.1234379

[gcbb12263-bib-0007] Bianchi F , Booij C , Tscharntke T (2006) Sustainable pest regulation in agricultural landscapes: a review on landscape composition, biodiversity and natural pest control. Proceedings of the Royal Society B: Biological Sciences, 273, 1715–1727. 16790403 10.1098/rspb.2006.3530 PMC1634792

[gcbb12263-bib-0008] Blanco‐Canqui H (2010) Energy crops and their implications on soil and environment. Agronomy Journal, 102, 403–419.

[gcbb12263-bib-0009] Boardman J , Poesen J (2006) Soil Erosion in Europe. Wiley Online Library, Chichester, UK.

[gcbb12263-bib-0010] Börjesson P (1999) Environmental effects of energy crop cultivation in sweden—i: Identification and quantification. Biomass and Bioenergy, 16, 137–154.

[gcbb12263-bib-0011] Borzecka‐Walker M , Faber A , Borek R (2008) Evaluation of carbon sequestration in energetic crops (Miscanthus and coppice willow). International Agrophysics, 22, 185–190.

[gcbb12263-bib-0012] Bosatta E , Agren GI (1991) Dynamics of carbon and nitrogen in the organic‐matter of the soil – a generic theory. American Naturalist, 138, 227–245.

[gcbb12263-bib-0013] Bourke D , Stanley D , O'rourke E *et al* (2014) Response of farmland biodiversity to the introduction of bioenergy crops: effects of local factors and surrounding landscape context. GCB Bioenergy, 6, 275–289.

[gcbb12263-bib-0014] Busch G (2012) Gis‐based tools for regional assessments and planning processes regarding potential environmental effects of poplar src. BioEnergy Research, 5, 584–605.

[gcbb12263-bib-0015] Clifton‐Brown JC , Breuer J , Jones MB (2007) Carbon mitigation by the energy crop, Miscanthus. Global Change Biology, 13, 2296–2307.

[gcbb12263-bib-0016] Clifton‐Brown J , Hastings A , Smith P , Stampfl P , Valentine J , Jones M , Donnison I (2008) Bioenergy technology—balancing energy output with environmental benefits. Comparative Biochemistry and Physiology Part A: Molecular & Integrative Physiology, 150, S174–S175.

[gcbb12263-bib-0017] Coleman K , Jenkinson D (1999) Rothc‐26.3. A Model for the Turnover of Carbon in Soil. Model Description and Windows Users Guide. University Press, Harpenden.

[gcbb12263-bib-0018] Dauber J , Jones MB , Stout JC (2010) The impact of biomass crop cultivation on temperate biodiversity. GCB Bioenergy, 2, 289–309.

[gcbb12263-bib-0019] Davis SC , Boddey RM , Alves BJ *et al* (2013) Management swing potential for bioenergy crops. GCB Bioenergy, 5, 623–638.

[gcbb12263-bib-0020] DEFRA (2013) Area of crops grown for bioenergy in England and the UK: 2008–2011, Experimental statistics, Non‐food crop areas – statistics notice (ed. Department for Environment Food & Rural Affairs), Government Statistical Service, pp. 1–32. Available at: http://www.defra.gov.uk/statistics/.

[gcbb12263-bib-0021] Dockerty T , Appleton K , Lovett A (2012) Public opinion on energy crops in the landscape: considerations for the expansion of renewable energy from biomass. Journal of Environmental Planning and Management, 55, 1134–1158.

[gcbb12263-bib-0022] Dondini M , Hastings A , Saiz G , Jones MB , Smith P (2009) The potential of Miscanthus to sequester carbon in soils: comparing field measurements in carlow, ireland to model predictions. GCB Bioenergy, 1, 413–425.

[gcbb12263-bib-0023] Donnelly A , Styles D , Fitzgerald J , Finnan J (2011) A proposed framework for determining the environmental impact of replacing agricultural grassland with Miscanthus in ireland. GCB Bioenergy, 3, 247–263.

[gcbb12263-bib-0024] Eigenbrod F , Bell V , Davies H , Heinemeyer A , Armsworth P , Gaston K (2011) The impact of projected increases in urbanization on ecosystem services. Proceedings of the Royal Society B: Biological Sciences, 278, 3201–3208. 21389035 10.1098/rspb.2010.2754 PMC3169018

[gcbb12263-bib-0025] European Commission (2012) Directive of the european parliament and of the council‐ amending directive 98/70/ec relating to the quality of petrol and diesel fuels and amending directive 2009/28/ec on the promotion of the use of energy from renewable sources (ed) 2012/0288 (COD).

[gcbb12263-bib-0026] FAO/Iiasa/ISRIC/Isscas/JRC (2009) Harmonized World Soil Database (Version 1.1), (ed. FAO R.), Italy and Iiasa, Laxenburg, Austria.

[gcbb12263-bib-0027] Fargione J , Hill J , Tilman D , Polasky S , Hawthorne P (2008) Land clearing and the biofuel carbon debt. Science, 319, 1235–1238. 18258862 10.1126/science.1152747

[gcbb12263-bib-0028] Finch JW , Riche AB (2010) Interception losses from Miscanthus at a site in south‐east england—an application of the gash model. Hydrological Processes, 24, 2594–2600.

[gcbb12263-bib-0029] Godfray HCJ , Beddington JR , Crute IR *et al* (2010) Food security: the challenge of feeding 9 billion people. science, 327, 812–818. 20110467 10.1126/science.1185383

[gcbb12263-bib-0030] Hansen EM , Christensen BT , Jensen L , Kristensen K (2004) Carbon sequestration in soil beneath long‐term *Miscanthus* plantations as determined by ^13^c abundance. Biomass and Bioenergy, 26, 97–105.

[gcbb12263-bib-0031] Hastings A , Clifton‐Brown J , Wattenbach M , Stampfl P , Mitchell CP , Smith P (2008) Potential of Miscanthus grasses to provide energy and hence reduce greenhouse gas emissions. Agronomy for sustainable development, 28, 465–472.

[gcbb12263-bib-0032] Hastings A , Clifton‐Brown J , Wattenbach M , Mitchell C , Smith P (2009a) The development of miscanfor, a new Miscanthus crop growth model: Towards more robust yield predictions under different climatic and soil conditions. GCB Bioenergy, 1, 154–170.

[gcbb12263-bib-0033] Hastings A , Clifton‐Brown J , Wattenbach M , Mitchell C , Stampfl P , Smith P (2009b) Future energy potential of Miscanthus in europe. GCB Bioenergy, 1, 180–196.

[gcbb12263-bib-0034] Hastings A , Tallis MJ , Casella E *et al* (2014) The technical potential of great britain to produce ligno‐cellulosic biomass for bioenergy in current and future climates. GCB Bioenergy, 6, 108–122.

[gcbb12263-bib-0035] Hillier J , Whittaker C , Dailey G *et al* (2009) Greenhouse gas emissions from four bioenergy crops in england and wales: Integrating spatial estimates of yield and soil carbon balance in life cycle analyses. GCB Bioenergy, 1, 267–281.

[gcbb12263-bib-0036] Holland RA , Eigenbrod F , Muggeridge A , Brown G , Clarke D , Taylor G (2015) A synthesis of the ecosystem services impact of second generation bioenergy production. Renewable & Sustainable Energy Reviews, 46, 30–40.

[gcbb12263-bib-0037] Jenkins G , Murphy J , Sexton D , Lowe J , Jones P , Kilsby C (2009) UK climate projections: Briefing report. Met office hadley centre, Exeter, UK. ISBN 978‐1‐906360‐04‐7. Available at: http://ukclimateprojections.defra.gov.uk (accessed 23 March 2015).

[gcbb12263-bib-0038] Kang S , Post WM , Nichols JA , Wang D , West TO , Bandaru V , Izaurralde RC (2013) Marginal lands: Concept, assessment and management. Journal of Agricultural Science, 5, p129.

[gcbb12263-bib-0039] Lattimore B , Smith C , Titus B , Stupak I , Egnell G (2009) Environmental factors in woodfuel production: opportunities, risks, and criteria and indicators for sustainable practices. Biomass and Bioenergy, 33, 1321–1342.

[gcbb12263-bib-0040] Le PV , Kumar P , Drewry DT (2011) Implications for the hydrologic cycle under climate change due to the expansion of bioenergy crops in the midwestern United States. Proceedings of the National Academy of Sciences of the United States of America, 108, 15085–15090. 21876137 10.1073/pnas.1107177108 PMC3174653

[gcbb12263-bib-0041] Lovett AA , Sünnenberg GM , Richter GM , Dailey AG , Riche AB , Karp A (2009) Land use implications of increased biomass production identified by gis‐based suitability and yield mapping for Miscanthus in england. BioEnergy Research, 2, 17–28.

[gcbb12263-bib-0042] Lovett A , Sünnenberg G , Dockerty T (2014) The availability of land for perennial energy crops in Great Britain. GCB Bioenergy, 6, 99–107.

[gcbb12263-bib-0043] Manning P , Taylor G , Hanley ME (2014) Bioenergy, food security and biodiversity – an unlikely alliance? GCB Bioenergy, Accepted article (Available at: http://onlinelibrary.wiley.com/doi/10.1111/gcbb.12173/abstract) (accessed 23 March 2015).

[gcbb12263-bib-0044] Matthews R , Grogan P , Bullard M , Christian D , Knight J , Lainsbury M , Parker S (2001) Potential c‐sequestration rates under short‐rotation coppiced willow and Miscanthus biomass crops: a modelling study. Aspects of Applied Biology, 65, 303–312.

[gcbb12263-bib-0045] Metzger M , Rounsevell M , Acosta‐Michlik L , Leemans R , Schröter D (2006) The vulnerability of ecosystem services to land use change. Agriculture, Ecosystems & Environment, 114, 69–85.

[gcbb12263-bib-0046] Millennium Ecosystem Assessment (2005) Ecosystems and human well‐being: Our human planet: Summary for decision makers, Island Pr.

[gcbb12263-bib-0047] Plevin RJ , Jones AD , Torn MS , Gibbs HK (2010) Greenhouse gas emissions from biofuels' indirect land use change are uncertain but may be much greater than previously estimated. Environmental science & technology, 44, 8015–8021. 20942480 10.1021/es101946t

[gcbb12263-bib-0048] Pyatt G , Ray D , Fletcher D (2001) An ecological site classification for forestry in great britain. Bulletin 124, Forestry Commission, Edinburgh.

[gcbb12263-bib-0049] Rathmann R , Szklo A , Schaeffer R (2010) Land use competition for production of food and liquid biofuels: an analysis of the arguments in the current debate. Renewable Energy, 35, 14–22.

[gcbb12263-bib-0050] Rowe RL , Street NR , Taylor G (2009) Identifying potential environmental impacts of large‐scale deployment of dedicated bioenergy crops in the UK. Renewable and sustainable energy reviews, 13, 271–290.

[gcbb12263-bib-0051] Rowe RL , Hanley ME , Goulson D , Clarke DJ , Doncaster CP , Taylor G (2011) Potential benefits of commercial willow short rotation coppice (src) for farm‐scale plant and invertebrate communities in the agri‐environment. Biomass and Bioenergy, 35, 325–336.

[gcbb12263-bib-0052] Rowe RL , Goulson D , Doncaster CP , Clarke DJ , Taylor G , Hanley ME (2013) Evaluating ecosystem processes in willow short rotation coppice bioenergy plantations. GCB Bioenergy, 5, 257–266.

[gcbb12263-bib-0501] Schulze ED , Korner CI , Law BE , Haberl H , Luyssaert S (2012) Large‐scale bioenergy from additional harvest of forest biomass is neither sustainable nor greenhouse gas neutral. Global Change Biology Bioenergy, 4, 611–616.

[gcbb12263-bib-0053] Searchinger T , Heimlich R , Houghton RA *et al* (2008) Use of us croplands for biofuels increases greenhouse gases through emissions from land‐use change. Science, 319, 1238–1240. 18258860 10.1126/science.1151861

[gcbb12263-bib-0054] Selman P (2010) Learning to love the landscapes of carbon‐neutrality. Landscape Research, 35, 157–171.

[gcbb12263-bib-0055] Semere T , Slater F (2007a) Ground flora, small mammal and bird species diversity in miscanthus (*Miscanthus* × *giganteus)* and reed canary‐grass (*phalaris arundinacea*) fields. Biomass and Bioenergy, 31, 20–29.

[gcbb12263-bib-0056] Semere T , Slater F (2007b) Invertebrate populations in miscanthus (*Miscanthus* × *giganteus*) and reed canary‐grass (*phalaris arundinacea*) fields. Biomass and Bioenergy, 31, 30–39.

[gcbb12263-bib-0057] Smith P , Martino D , Cai Z *et al* (2008) Greenhouse gas mitigation in agriculture. Philosophical Transactions of the Royal Society B: Biological Sciences, 363, 789–813. 10.1098/rstb.2007.2184 PMC2610110 17827109

[gcbb12263-bib-0058] St Clair S , Hillier J , Smith P (2008) Estimating the pre‐harvest greenhouse gas costs of energy crop production. Biomass and Bioenergy, 32, 442–452.

[gcbb12263-bib-0059] Tallis MJ , Casella E , Henshall PA , Aylott MJ , Randle TJ , Morison JIL , Taylor G (2013) Development and evaluation of forestgrowth‐src a process‐based model for short rotation coppice yield and spatial supply reveals poplar uses water more efficiently than willow. GCB Bioenergy, 5, 53–66.

[gcbb12263-bib-0060] Thompson DA , Matthews RW (1989) The storage of carbon in trees and timber. Research Information Note‐Forestry Commission Research Division,160.

[gcbb12263-bib-0061] Tilman D , Socolow R , Foley JA *et al* (2009) Beneficial biofuels—the food, energy, and environment trilemma. Science, 325, 270. 19608900 10.1126/science.1177970

[gcbb12263-bib-0062] Tirado M , Clarke R , Jaykus L , Mcquatters‐Gollop A , Frank J (2010) Climate change and food safety: a review. Food Research International, 43, 1745–1765.

[gcbb12263-bib-0063] UK National Ecosystem Assessment (2011) The UK National Ecosystem Assessment Technical Report. UNEP‐WCMC, Cambridge.

[gcbb12263-bib-0064] Updegraff K , Baughman MJ , Taff SJ (2004) Environmental benefits of cropland conversion to hybrid poplar: economic and policy considerations. Biomass and Bioenergy, 27, 411–428.

[gcbb12263-bib-0065] Upham P , Shackley S (2007) Local public opinion of a proposed 21.5 mw (e) biomass gasifier in devon: Questionnaire survey results. Biomass and Bioenergy, 31, 433–441.

[gcbb12263-bib-0066] Valentine J , Clifton‐Brown J , Hastings A , Robson P , Allison G , Smith P (2012) Food vs. Fuel: the use of land for lignocellulosic ‘next generation'energy crops that minimize competition with primary food production. GCB Bioenergy, 4, 1–19.

[gcbb12263-bib-0067] Vanloocke A , Bernacchi CJ , Twine TE (2010) The impacts of Miscanthus× giganteus production on the midwest us hydrologic cycle. GCB Bioenergy, 2, 180–191.

[gcbb12263-bib-0068] Wang S , Hastings A , Smith P (2012) An optimization model for energy crop supply. GCB Bioenergy, 4, 88–95.

[gcbb12263-bib-0069] Wang S , Hastings A , Wang S *et al* (2014) The potential for bioenergy crops to contribute to meeting gb heat and electricity demands. GCB Bioenergy, 6, 136–141.

[gcbb12263-bib-0070] Werling BP , Dickson TL , Isaacs R *et al* (2014) Perennial grasslands enhance biodiversity and multiple ecosystem services in bioenergy landscapes. Proceedings of the National Academy of Sciences of the United States of America, 111, 1652–1657. 24474791 10.1073/pnas.1309492111 PMC3910622

[gcbb12263-bib-0071] Whitaker J , Ludley KE , Rowe R , Taylor G , Howard DC (2010) Sources of variability in greenhouse gas and energy balances for biofuel production: a systematic review. GCB Bioenergy, 2, 99–112.

[gcbb12263-bib-0072] Wynn JG , Bird MI (2007) C4‐derived soil organic carbon decomposes faster than its C3 counterpart in mixed C3/C4 soils. Global Change Biology, 13, 2206–2217.

[gcbb12263-bib-0073] Yan X , Inderwildi OR , King DA (2010) Biofuels and synthetic fuels in the us and china: a review of well‐to‐wheel energy use and greenhouse gas emissions with the impact of land‐use change. Energy & Environmental Science, 3, 190–197.

[gcbb12263-bib-0074] Zatta A , Clifton‐Brown J , Robson P , Hastings A , Monti A (2014) Land use change from C3 grassland to C4 Miscanthus: effects on soil carbon content and estimated mitigation benefit after six years. GCB Bioenergy, 6, 360–370.

[gcbb12263-bib-0075] Zimmermann J , Dauber J , Jones MB (2012) Soil carbon sequestration during the establishment phase of miscanthus × giganteus: a regional‐scale study on commercial farms using 13c natural abundance. GCB Bioenergy, 4, 453–461.

