# Supplementary material for: Potential impacts on ecosystem services of land use transitions to second‐generation bioenergy crops in GB
Source: Glob Change Biol Bioenergy. 2015 Jun 8;8(2):317–33. doi: 10.1111/gcbb.12263 (PMC4974899; doi:10.1111/gcbb.12263)
Supplement: Supplementary file 1 — Appendix S1. Detailed approach taken to compile the ecosystem service impact matrix. Figure S1. Flow chart of steps taken in compiling threat matrix. Table S1. Results of literature review indicating ecosystem services examined, keywords used in the Web of Science search and the total references after each of the filtering criteria were applied. Table S2. Studies that use a reference state approach to examine the implications of transitions to 2G bioenergy feedstocks. [file GCBB-8-317-s001.docx]

**Supplementary Online Material – Ecosystem service impact matrix**

**Approach**

Full details of the methodology and results of the literature search are provided in (Holland *et al.*, 2015). However, to aid the reader we provide a brief description of the method and key references below.

Searches were performed in Web of Science (WoS) during December 2014 using the ecosystem service keywords detailed in Table S1 together with ‘biofuel’, ‘biodiesel’, ‘bioethanol’, and ‘bioenergy’. In total the WoS search returned 8,743 references that were then assessed for relevance as described below and detailed in Figure S1.

At the first stage the 8,743 references were filtered based on their title and abstract. The full text of the remaining 681 references were obtained were possible and a second stage of filtering carried out to identify those that considered relevant bioenergy feedstock and land use transitions based on a reference state approach. Here studies were retained if they considered a temporal change in the provision of ecosystem services during conversion of an area from one of our defined reference states to 2G feedstock production. Alternatively, studies that used a space for time substitution comparing ecosystem service provision under a reference state to provision under 2G feedstock production using comparable areas in close proximity. Studies that qualified under this criteria are listed in Table S2.

For each study we captured the ecosystem service examined, the specific feedstock, the geographic location, the land-use transition and whether the study used empirical data collected in the field or was based on a modelling approach (see Table S2). Transitions were scored as having a positive, negative or neutral effect on an ecosystem service based on the statistical analysis presented in the study and the stated results and conclusions of the authors. We do not present specific data from each study as it was not possible to extract mean values and standard errors for each effect as these were not always stated by the authors, and extracting the data from presented figures would introduce error. Furthermore, in contrast to greenhouse gas emissions where it is often possible to standardise results across studies into a common unit, the diverse set of measures used in the studies presented in Table S2 prevent meaningful comparison based on standard units in all but a few instances. Where more than one feedstock, ecosystem service, geographic location or land use transition was considered we disaggregated the results to capture individual reported effects. Land-use transition were initially classified studies based on three categories, arable land, marginal and forest. Based on detailed site descriptions from the text those initially classified as marginal land were further subdivided into studies to identify those that dealt specifically with grassland systems.

As noted by Donnelly *et al.* (2011) there remains significant gaps in our understanding of the implications of transitions to 2G feedstock production for many ecosystem services, even those that are areas of significant research interest such as greenhouse gas emissions (Anderson-Teixeira *et al.*, 2009; Cherubini *et al.*, 2009; Hillier *et al.*, 2009; Drewer *et al.*, 2012) and biodiversity (Rowe *et al.*, 2009; Dauber *et al.*, 2010; Fletcher *et al.*, 2011).

The studies detailed in Table S2 were supplemented with other empirical studies and reviews returned during our Web of Science search that did not use a reference state approach but are relevant to the ecosystem services and transition examined (Figure S1).

Finally, in interpreting implications of land use transitions there are two general points that were incorporated into the assessment presented in Table 1. Firstly, across studies the length of the management cycle emerges as key to understanding the implications of transitions to 2G feedstock production (Lattimore *et al.*, 2009; Donnelly *et al.*, 2011; Schulze *et al.*, 2012). As such, and in the absence of evidence to the contrary, it would be expected that most benefits are realised through conversion of arable land to 2G feedstock production, fewer benefits for conversion of marginal or grassland, and the least benefits for conversion from forest or woodland.

Secondly, there are strong cross linkages between different ecosystem services such that mechanisms relevant for impacts on one will also influence our understanding of impacts on the provision of other ecosystem services. For example changes in Soil Quality associated with land use transitions to 2G feedstock production (Börjesson, 1999; Blanco-Canqui, 2010; Haney *et al.*, 2010; Donnelly *et al.*, 2011; Pellegrino *et al.*, 2011) will also deliver benefits for the service of Hazard Regulation through the control of surface runoff (Blanco-Canqui, 2010) and wind erosion (Busch, 2012).

**Hazard Regulation**

In addition to those studies presented in Table S2 our assessment was informed by a number of reviews (Börjesson, 1999; Donnelly *et al.*, 2011) and studies (Updegraff *et al.*, 2004; Boardman & Evans, 2006; Lattimore *et al.*, 2009; Busch, 2012) that consider the role of agricultural practices in influencing the provision of this service, particularly in relation to the establishment and harvesting phases (Lattimore *et al.*, 2009; Donnelly *et al.*, 2011). Of particular relevance is the influence of 2G feedstocks on soil properties (see Soil Quality below), that promotes a reduction in surface runoff (Blanco-Canqui, 2010) and wind erosion (Busch, 2012).

**Disease and Pest control**

In addition to those studies presented in Table S2 (Gardiner *et al.*, 2010; Werling *et al.*, 2011a, 2014), a number of empirical studies and reviews (Sage, 1998; Rowe *et al.*, 2009, 2011; Dauber *et al.*, 2010; Power, 2010; Thomson & Hoffmann, 2011; Bourke *et al.*, 2014) examining biodiversity associated with 2G feedstocks have reported enhancement of predators and parasitoids including arthropods, birds, mammals and microbial pathogens. Although the exact relationship between biodiversity and disease and pest control is contested (Myers *et al.*, 1989; Bianchi *et al.*, 2006; Cardinale *et al.*, 2006) beneficial effects for biodiversity associated with transitions to 2G feedstock production would likely enhance this service through a range of mechanisms.

**Pollination**

Changes in management practices, vegetation and habitat structure and diversity associated with land use transition to 2G feedstocks have been demonstrated to either enhance or be neutral for pollinators and pollination services depending on the specific transition (Carvell *et al.*, 2007; Holzschuh *et al.*, 2007; Klein *et al.*, 2007; Kremen *et al.*, 2007; Rowe *et al.*, 2011; Stanley & Stout, 2013). However, due to limited large scale deployment of 2G feedstock it is unclear whether loss of habitat diversity at commercial scales may lead to a reduction in these benefits (Dahms *et al.*, 2010; Dauber *et al.*, 2010; Gardiner *et al.*, 2010; Robertson *et al.*, 2012) and so impact this service.

**Soil Quality**

Of particular interest, due to its relationship with greenhouse gas emissions, are changes in SOC which have been investigated in detail through studies that considered specific transitions of relevance in Ireland (e.g. (Zimmermann *et al.*, 2012, 2013)) and the UK (e.g.(Keith *et al.*, 2014)) together with the review of (Don *et al.*, 2012). As detailed in the main text changes in SOC were modelled in the current study, however we incorporated these results with a consideration of other aspects of soil quality.

A number of studies and reviews (Börjesson, 1999; Blanco-Canqui, 2010; Haney *et al.*, 2010; Donnelly *et al.*, 2011; Pellegrino *et al.*, 2011) suggest transitions to 2G feedstocks can deliver a range of benefits including reduced bulk density, improved soil porosity, improved microbial activity and biomass, improved macro-invertebrate populations, and improved fluxes of water, air and heat. These in turn contribute to changes in delivery of other services such as Hazard Regulation. There may be distinct temporal patterns in the delivery of this services with certain phases in feedstock production (establishment, harvesting) having a short lived but negative impact on soil quality (Lattimore *et al.*, 2009; Donnelly *et al.*, 2011).

**Water Quality**

In addition to those studies identified in Table S2 and reviews of Börjesson (1999) and Donnelly *et al.* (2011), a number of studies were identified that specifically examined fertiliser application rates and impacts on water quality associated with transitions to 2G feedstocks. Benefits were associated with reduced rates of fertiliser application for 2G feedstocks (Ng *et al.*, 2010; Callesen *et al.*, 2011; Dimitriou *et al.*, 2012; Gonzalez-Garcia *et al.*, 2012), although studies suggest there may be a trade-off with yield (Ng *et al.*, 2010; Gonzalez-Garcia *et al.*, 2012). As with Soil Quality (see above) distinct temporal patterns were noted with impacts on water quality associated with establishment and harvesting phase (Lattimore *et al.*, 2009; Donnelly *et al.*, 2011; Dimitriou *et al.*, 2012; Gonzalez-Garcia *et al.*, 2012; Palmer *et al.*, 2014) and timing of agrochemical application (Love & Nejadhashemi, 2011; Syswerda *et al.*, 2012).

**Biodiversity**

A number of meta-analyses have considered implications of bioenergy crops for biodiversity (Rowe *et al.*, 2009; Dauber *et al.*, 2010; Fletcher *et al.*, 2011) so we did not include this as a search term in our literature review, but drew upon the conclusions of these authors. These studies were supplemented with subsequent empirical studies that considered relevant land use transitions (Landis & Werling, 2010; Stanley & Stout, 2013; Bourke *et al.*, 2014) and other relevant reviews (Börjesson, 1999; Donnelly *et al.*, 2011).

**Food and Fibre**

Although the production of bioenergy feedstocks fall within this category, we restrict our consideration to other crop and livestock production. In addition to those studies described in Table S2 studies by Lovett *et al.* (2009) and Aylott *et al.* (2010) suggest that in the UK there is considerable scope for expansion of 2G feedstock production with little effect on food production through the utilisation of marginal land. This contrasts with other studies that suggest that the importance of marginal lands could be underestimated (Kang *et al.*, 2013) leading to direct competition between 2G feedstock production and traditional forage-livestock production (Sanderson & Adler, 2008).

**Timber and Forest Products**

As arable land and Improved and Semi Improved grassland currently do not produce timber or forest products these transition were scored as neutral. Our literature review identified no studies that considered transitions of land currently utilised for forestry or as a woodland to Miscanthus within Europe or the US, and it is unlikely that large scale transitions will occur particularly in Europe where policy is designed to increase afforestation. Two studies (see Table S2) considered transitions in developing countries and highlighting social aspects. Conversion of existing plantation forestry to SRC and SRF for 2G feedstock production represents the most likely pathway that will influence the provision of timber and forest products, although economic factors determining final use are likely to be key drivers of impacts and not a reduction in total available timber and forest products (Kirilenko & Sedjo, 2007; Raunikar *et al.*, 2010).

**Water Availability**

There is relatively little research on the effects of 2G feedstocks on water availability (Donnelly *et al.*, 2011). In addition to those studies presented in Table S2, our assessment is based on studies of crop characteristics (Finch & Riche, 2010; Hickman *et al.*, 2010; Le *et al.*, 2011; VanLoocke *et al.*, 2012), and landscape scale patterns, with factors such as seasonal water availability (Lattimore *et al.*, 2009; Oliver *et al.*, 2009; Stone *et al.*, 2010) and economically realistic deployment strategies (Vanloocke *et al.*, 2010; Kocoloski *et al.*, 2011) being significant drivers of impacts.

**Food from Marine Ecosystems**

We identified no studies that examined how the land use transitions identified in Table 1 influence the provision of this service. However, improvements in water quality (see section above) associated particularly with transition from arable production could help ameliorate seasonal hypoxic zones that are a significant threat to fisheries globally (Diaz & Rosenberg, 1995; Alexander *et al.*, 2000; Goolsby & Battaglin, 2000; Diaz, 2001).

**Game and Wild Food**

We identified no studies that specifically examined this service. Game species are often managed due to the revenue that they can generate. For terrestrial species, such as pheasant and deer, this management will probably mean that bioenergy production will have little influence on the provision of the service.

**Honey**

We identified no studies that specifically examined honey production, however we refer the reader to references contained within the section on biodiversity relating to implications for pollinator species.

**Ornamental Resources**

The UK National Ecosystem Assessment (2011) reports that in 2002 (the latest date for which data is available) production of flowers in the UK was worth £674 million annually. As with Food and Fibre, and Timber and Forest services the principal threat is competition for land.

**Genetic Resources**

The UK National Ecosystem Assessment (2011) considers that genetic resources are explicitly linked to biodiversity. We therefore refer the reader to this section (see above).

**Table S1: Results of literature review indicating ecosystem services examined, keywords used in the Web of Science search and the total references after each of the filtering criteria were applied.** First filter refers to the initial review of papers based on title and abstract. Second filter refers to those studies that used a reference state approach to examine impacts. Numbers in parentheses are studies added subsequent to December 2012.

| \| Ecosystem Service \| Keywords \| Total Refs. \| 1st Filter \| 2nd Filter \| \| --- \| --- \| --- \| --- \| --- \| \| Hazard regulation \| Erosion; Flooding \| 152 \| 30 \| 11 \| \| Disease and Pest control \| Pest; Disease \| 200 \| 25 \| 7 \| \| Pollination \| Pollination; Pollinators; Bees \| 44 \| 21 \| 5 \| \| Soil quality \| Soil; Soil cycling; Nutrient cycling; Carbon; \| 4762 \| 251 \| 29 \| \| Water quality \| Eutrophication; Water quality \| 510 \| 76 \| 18 \| \| Food and Fibre \| Livestock; Food; Fibre; Pasture; Forage \| 1422 \| 112 \| 6 \| \| Timber and forest products \| Timber; Forest; Forestry; Fungi \| 1217 \| 92 \| 1 \| \| Water availability \| Water quantity; Water availability \| 214 \| 54 \| 5 \| \| Food from marine ecosystems \| Fisheries; Fish \| 166 \| 10 \| 0 \| \| Game and wild food \| Deer: Pheasant: Hunting; Fungi \| 0 \| 0 \| 0 \| \| Honey production \| Bees; Honey \| 23 \| 8 \| 0 \| \| Ornamental Resources \| Flowers; Horticulture \| 33 \| 2 \| 0 \| |  |  |
| --- | --- | --- | --- | --- | --- | --- | --- | --- | --- | --- | --- | --- | --- | --- | --- | --- | --- | --- | --- | --- | --- | --- | --- | --- | --- | --- | --- | --- | --- | --- | --- | --- | --- | --- | --- | --- | --- | --- | --- | --- | --- | --- | --- | --- | --- | --- | --- | --- | --- | --- | --- | --- | --- | --- | --- | --- | --- | --- | --- | --- | --- | --- | --- | --- | --- | --- | --- |
|  |  |  |
|  |  |  |
|  |  |  |
|  |  |  |
|  |  |  |
|  |  |  |
|  |  |  |
|  |  |  |
|  |  |  |
|  |  |  |
|  |  |  |
|  |  |  |

**Table S2: Studies that use a reference state approach to examine the implications of transitions to 2G bioenergy feedstocks.** In each case we indicate the country where the study was carried out, the ecosystem services considered, the reference land use and transition (whether to energy grasses, short rotation coppice or short rotation forestry), the specific crop identified in the study and whether the study was based on a modelling or experimental approach. In each case we indicate whether the effect identified was positive (+ve), negative (-ve) or neutral (Neu) in terms of impact on the provision of the service.

| Author | Country | Service | Reference land use | Transition land use | Crop specific | Modelling | +ve | -ve | Neu. |
| --- | --- | --- | --- | --- | --- | --- | --- | --- | --- |
| (Eller *et al.*, 2011) | USA | Air quality regulation | Arable | Grasses | Switchgrass | Experimental | 0 | 0 | 0 |
| (Monti *et al.*, 2009) | Global | Air quality regulation | Arable | Grasses | Cynara | Modelled | 1 | 0 | 0 |
| (Monti *et al.*, 2009) | Global | Air quality regulation | Arable | Grasses | Switchgrass | Modelled | 1 | 0 | 0 |
| (Monti *et al.*, 2009) | Global | Air quality regulation | Arable | Grasses | Miscanthus | Modelled | 1 | 0 | 0 |
| (Monti *et al.*, 2009) | Global | Air quality regulation | Arable | Grasses | Giant Reed | Modelled | 1 | 0 | 0 |
| (Ruiz-Valdiviezo *et al.*, 2010) | Mexico | Air quality regulation | Arable | Grasses | Jatropha | Experimental | 0 | 1 | 0 |
| (Toma *et al.*, 2011) | Japan | Air quality regulation | Grassland | Grasses | Miscanthus | Experimental | 1 | 0 | 0 |
| (Woli *et al.*, 2010) | USA | Air quality regulation | Arable | Grasses | Miscanthus | Experimental | 0 | 0 | 1 |
| (Woli *et al.*, 2010) | USA | Air quality regulation | Arable | Grasses | Switchgrass | Experimental | 0 | 0 | 1 |
| (Werling *et al.*, 2014) | USA | Disease and pest control | Arables | Grasses | Switchgrass | Experimental | 1 | 0 | 0 |
| (Werling *et al.*, 2014) | USA | Disease and pest control | Arables | Grasses | Prairie | Experimental | 1 | 0 | 0 |
| (Werling *et al.*, 2014) | USA | Disease and pest control | Arables | Grasses | Switchgrass | Experimental | 1 | 0 | 0 |
| (Werling *et al.*, 2014) | USA | Disease and pest control | Arables | Grasses | Prairie | Experimental | 1 | 0 | 0 |
| (Gardiner *et al.*, 2010) | USA | Disease and pest control | Arable | Grasses | Switchgrass | Experimental | 1 | 0 | 0 |
| (Gardiner *et al.*, 2010) | USA | Disease and pest control | Arable | Grasses | Mixed grasses | Experimental | 1 | 0 | 0 |
| (Werling *et al.*, 2011b) | USA | Disease and pest control | Arable | Grasses | Mixed grasses | Experimental | 1 | 0 | 0 |
| (Ariza-Montobbio & Lele, 2010) | India | Food and Fibre | Arable | Grasses | Jatropha | Experimental | 0 | 1 | 0 |
| (Davis *et al.*, 2012) | USA | Food and Fibre | Arable | Grasses | Switchgrass | Modelled | 1 | 0 | 0 |
| (Findlater & Kandlikar, 2011) | India | Food and Fibre | Marginal | Grasses | Jatropha | Experimental | 0 | 1 | 0 |
| (Garg *et al.*, 2011) | India | Food and Fibre | Marginal | Grasses | Jatropha | Modelled | 0 | 0 | 1 |
| (German *et al.*, 2011) | Zambia | Food and Fibre | Marginal | Grasses | Jatropha | Experimental | 0 | 1 | 0 |
| (Rittenburg *et al.*, 2011) | India | Food and Fibre | Arable | Grasses | Jatropha | Experimental | 0 | 1 | 0 |
| (Skutsch *et al.*, 2011) | Mexico | Food and Fibre | Forest | Grasses | Jatropha | Experimental | 0 | 0 | 1 |
| (Werling *et al.*, 2014) | USA | Food and Fibre | Arables | Grasses | Switchgrass | Experimental | 0 | 1 | 0 |
| (Werling *et al.*, 2014) | USA | Food and Fibre | Arables | Grasses | Prairie | Experimental | 0 | 1 | 0 |
| (Brown *et al.*, 2000) | USA | Hazard regulation | Arable | Grasses | Switchgrass | Modelled | 1 | 0 | 0 |
| (Evers *et al.*, 2013) | USA | Hazard regulation | Arable | Grasses | Switchgrass | Experimental | 1 | 0 | 0 |
| (Evers *et al.*, 2013) | USA | Hazard regulation | Arable | Grasses | Big Bluestem | Experimental | 1 | 0 | 0 |
| (Evers *et al.*, 2013) | USA | Hazard regulation | Arable | Grasses | Miscanthus | Experimental | 1 | 0 | 0 |
| (Garg *et al.*, 2011) | India | Hazard regulation | Marginal | Grasses | Jatropha | Modelled | 1 | 0 | 0 |
| (Wilson *et al.*, 2011) | USA | Hazard regulation | Arable | Grasses | Big Bluestem | Experimental | 1 | 0 | 0 |
| (Wilson *et al.*, 2011) | USA | Hazard regulation | Arable | Grasses | Smooth Bromegrass | Experimental | 1 | 0 | 0 |
| (Wilson *et al.*, 2011) | USA | Hazard regulation | Arable | Grasses | Switchgrass | Experimental | 1 | 0 | 0 |
| (Wu & Liu, 2012) | USA | Hazard regulation | Arable | Grasses | Miscanthus | Modelled | 1 | 0 | 0 |
| (Wu & Liu, 2012) | USA | Hazard regulation | Arable | Grasses | Switchgrass | Modelled | 1 | 0 | 0 |
| (Xue *et al.*, 2011) | USA | Hazard regulation | Grassland | Grasses | Mixed grasses | Experimental | 0 | 1 | 0 |
| (Christian *et al.*, 2006) | UK | Nutrient cycling | Arable | Grasses | Miscanthus | Experimental | 0 | 0 | 1 |
| (Haney *et al.*, 2010) | USA | Nutrient cycling | Arable | Grasses | Buffalo grass | Experimental | 1 | 0 | 0 |
| (Haney *et al.*, 2010) | USA | Nutrient cycling | Arable | Grasses | Switchgrass | Experimental | 1 | 0 | 0 |
| (Haney *et al.*, 2010) | USA | Nutrient cycling | Arable | Grasses | Bermuda Grass | Experimental | 1 | 0 | 0 |
| (Ruiz-Valdiviezo *et al.*, 2010) | Mexico | Nutrient cycling | Arable | Grasses | Jatropha | Experimental | 0 | 0 | 1 |
| (Gardiner *et al.*, 2010) | USA | Pollination | Arable | Grasses | Mixed grasses | Experimental | 1 | 0 | 0 |
| (Gardiner *et al.*, 2010) | USA | Pollination | Arable | Grasses | Switchgrass | Experimental | 1 | 0 | 0 |
| (Werling *et al.*, 2014) | USA | Pollination | Arables | Grasses | Prairie | Experimental | 1 | 0 | 0 |
| (Anderson-Teixeira *et al.*, 2009) | Global | Soil quality | Grassland | Grasses | Miscanthus | Modelled | 0 | 0 | 1 |
| (Anderson-Teixeira *et al.*, 2009) | Global | Soil quality | Grassland | Grasses | Switchgrass | Modelled | 0 | 0 | 1 |
| (Anderson-Teixeira *et al.*, 2009) | Global | Soil quality | Grassland | Grasses | Mixed grasses | Modelled | 1 | 0 | 0 |
| (Dowell *et al.*, 2009) | USA | Soil quality | Arable | SRC | Poplar | Experimental | 0 | 1 | 0 |
| (Evers *et al.*, 2013) | USA | Soil quality | Arable | Grasses | Switchgrass | Experimental | 0 | 0 | 1 |
| (Evers *et al.*, 2013) | USA | Soil quality | Arable | Grasses | Big Bluestem | Experimental | 0 | 0 | 1 |
| (Evers *et al.*, 2013) | USA | Soil quality | Arable | Grasses | Miscanthus | Experimental | 0 | 0 | 1 |
| (Garg *et al.*, 2011) | India | Soil quality | Marginal | Grasses | Jatropha | Modelled | 1 | 0 | 0 |
| (Gelfand *et al.*, 2011) | USA | Soil quality | Grassland | Grasses | Mixed | Modelled | 0 | 1 | 0 |
| (Haney *et al.*, 2010) | USA | Soil quality | Arable | Grasses | Buffalo grass | Experimental | 1 | 0 | 0 |
| (Haney *et al.*, 2010) | USA | Soil quality | Arable | Grasses | Bermuda Grass | Experimental | 1 | 0 | 0 |
| (Haney *et al.*, 2010) | USA | Soil quality | Arable | Grasses | Switchgrass | Experimental | 1 | 0 | 0 |
| (Keith *et al.*, 2014) | UK | Soil quality | Arables | SRF | SRF Coniferous | Experimental | 1 | 0 | 0 |
| (Keith *et al.*, 2014) | UK | Soil quality | Arables | SRF | Eucalyputs | Experimental | 0 | 0 | 1 |
| (Keith *et al.*, 2014) | UK | Soil quality | Arables | SRF | Broadleaved | Experimental | 0 | 0 | 1 |
| (Ma *et al.*, 2000) |  | Soil quality | Grassland | Grasses | Switchgrass | Experimental | 1 | 0 | 0 |
| (Ma *et al.*, 2000) | USA | Soil quality | Marginal | Grasses | Switchgrass | Experimental | 1 | 0 | 0 |
| (Ruiz-Valdiviezo *et al.*, 2010) | Mexico | Soil quality | Arable | Grasses | Jatropha | Experimental | 0 | 0 | 1 |
| (Schmer *et al.*, 2011) | USA | Soil quality | Arable | Grasses | Switchgrass | Experimental | 0 | 0 | 1 |
| (Zimmermann *et al.*, 2013) | Ireland | Soil quality | Arable | Grasses | Miscanthus | Experimental | 0 | 0 | 1 |
| (Zimmermann *et al.*, 2013) | Ireland | Soil quality | Grassland | Grasses | Miscanthus | Experimental | 0 | 0 | 1 |
| (German *et al.*, 2011) | Zambia | Timber and forest products | Marginal | Grasses | Jatropha | Experimental | 0 | 1 | 0 |
| (Skutsch *et al.*, 2011) | Mexico | Timber and forest products | Forest | Grasses | Jatropha | Experimental | 0 | 0 | 1 |
| (Brown *et al.*, 2000) | USA | Water availability | Arable | Grasses | Switchgrass | Modelled | 0 | 1 | 0 |
| (Garg *et al.*, 2011) | India | Water availability | Marginal | Grasses | Jatropha | Modelled | 0 | 1 | 0 |
| (Hickman *et al.*, 2010) |  | Water availability | Arable | Grasses | Miscanthus | Experimental | 0 | 1 | 0 |
| (Wu & Liu, 2012) | USA | Water availability | Arable | Grasses | Switchgrass | Modelled | 0 | 1 | 0 |
| (Wu & Liu, 2012) | USA | Water availability | Arable | Grasses | Miscanthus | Modelled | 0 | 1 | 0 |
| (Davis *et al.*, 2012) | USA | Water quality | Arable | Grasses | Switchgrass | Modelled | 1 | 0 | 0 |
| (Love *et al.*, 2011) | USA | Water quality | Arable | Grasses | Switchgrass | Modelled | 1 | 0 | 0 |
| (Monti *et al.*, 2009) | Global | Water quality | Arable | Grasses | Giant Reed | Modelled | 1 | 0 | 0 |
| (Monti *et al.*, 2009) | Global | Water quality | Arable | Grasses | Cynara | Modelled | 1 | 0 | 0 |
| (Monti *et al.*, 2009) | Global | Water quality | Arable | Grasses | Miscanthus | Modelled | 1 | 0 | 0 |
| (Monti *et al.*, 2009) | Global | Water quality | Arable | Grasses | Switchgrass | Modelled | 1 | 0 | 0 |
| (Syswerda *et al.*, 2012) | USA | Water quality | Forest | Grasses | Alfafa | Experimental | 0 | 1 | 0 |
| (Syswerda *et al.*, 2012) | USA | Water quality | Forest | SRC | Poplar | Experimental | 1 | 0 | 0 |
| (Wilson *et al.*, 2011) | USA | Water quality | Arable | Grasses | Switchgrass | Experimental | 1 | 0 | 0 |
| (Wilson *et al.*, 2011) | USA | Water quality | Arable | Grasses | Big Bluestem | Experimental | 1 | 0 | 0 |
| (Wilson *et al.*, 2011) | USA | Water quality | Arable | Grasses | Smooth Bromegrass | Experimental | 1 | 0 | 0 |
| (Wu & Liu, 2012) | USA | Water quality | Arable | Grasses | Miscanthus | Modelled | 0 | 1 | 0 |
| (Wu & Liu, 2012) | USA | Water quality | Arable | Grasses | Switchgrass | Modelled | 0 | 1 | 0 |

**Figure S1: Flow chart of steps taken in compiling threat matrix.**

**
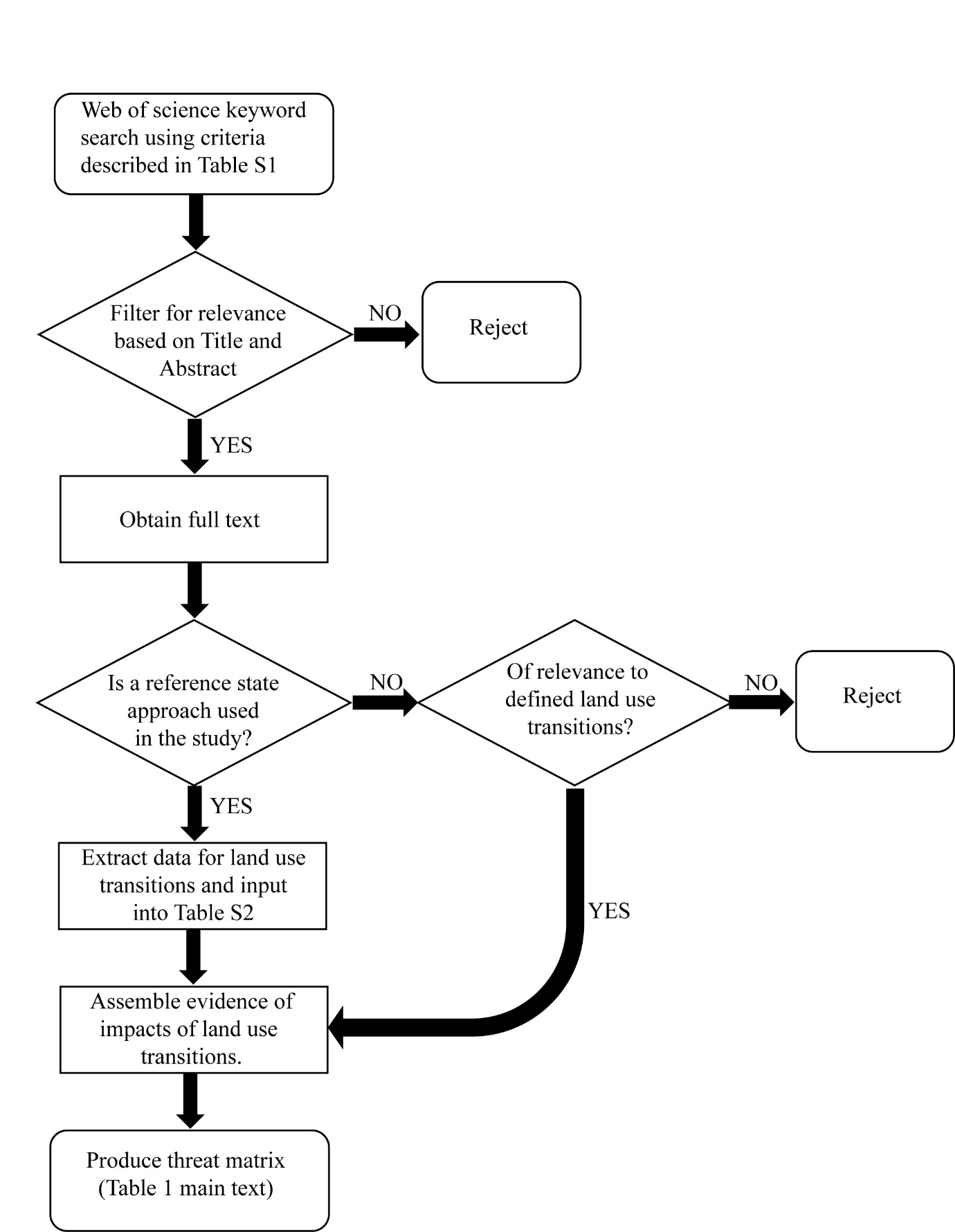
**

**Supplementary References**

Alexander RB, Smith RA, Schwarz GE (2000) Effect of stream channel size on the delivery of nitrogen to the Gulf of Mexico. *Nature*, **403**, 758–761.

Anderson-Teixeira KJ, Davis SC, Masters MD, Delucia EH (2009) Changes in soil organic carbon under biofuel crops. *Global Change Biology Bioenergy*, **1**, 75–96.

Ariza-Montobbio P, Lele S (2010) Jatropha plantations for biodiesel in Tamil Nadu, India Viability, livelihood trade-offs, and latent conflict. *Ecological Economics*, **70**, 189–195.

Aylott MJ, Casella E, Farrall K, Taylor G (2010) Estimating the supply of biomass from short-rotation coppice in England, given social, economic and environmental constraints to land availability. *Biofuels Bioproducts & Biorefining-Biofpr*, **5**, 719–727.

Bianchi FJJA, Booij CJH, Tscharntke T (2006) Sustainable pest regulation in agricultural landscapes: a review on landscape composition, biodiversity and natural pest control. *Proceedings of the Royal Society B-Biological Sciences*, **273**, 1715–1727.

Blanco-Canqui H (2010) Energy Crops and Their Implications on Soil and Environment. *Agronomy Journal*, **102**, 403–419.

Boardman J, Evans R (2006) Britain. In: *Soil Erosion in Europe* (eds Boardman J, Poesen J). John Wiley & Sons, Chichester, UK.

Börjesson P (1999) Environmental effects of energy crop cultivation in Sweden—I: Identification and quantification. *Biomass and Bioenergy*, **16**, 137–154.

Bourke D, Stanley D, O’Rourke E et al. (2014) Response of farmland biodiversity to the introduction of bioenergy crops: effects of local factors and surrounding landscape context. *GCB Bioenergy*, **6**, 275–289.

Brown RA, Rosenberg NJ, Hays CJ, Easterling WE, Mearns LO (2000) Potential production and environmental effects of switchgrass and traditional crops under current and greenhouse-altered climate in the central United States: a simulation study. *Agriculture Ecosystems & Environment*, **78**, 31–47.

Busch G (2012) GIS-based Tools for Regional Assessments and Planning Processes Regarding Potential Environmental Effects of Poplar SRC. *Bioenergy Research*, **5**, 584–605.

Callesen I, Carter MS, Ostergard H (2011) Efficient use of reactive nitrogen for cultivation of bioenergy: less is more. *Global Change Biology Bioenergy*, **3**, 171–179.

Cardinale BJ, Srivastava DS, Duffy JE, Wright JP, Downing AL, Sankaran M, Jouseau C (2006) Effects of biodiversity on the functioning of trophic groups and ecosystems. *Nature*, **443**, 989–92.

Carvell C, Meek WR, Pywell RF, Goulson D, Nowakowski M (2007) Comparing the efficacy of agri-environment schemes to enhance bumble bee abundance and diversity on arable field margins. *Journal of Applied Ecology*, **44**, 29–40.

Cherubini F, Bird ND, Cowie A, Jungmeier G, Schlamadinger B, Woess-Gallasch S (2009) Energy- and greenhouse gas-based LCA of biofuel and bioenergy systems: Key issues, ranges and recommendations. *Resources Conservation and Recycling*, **53**, 434–447.

Christian DG, Poulton PR, Riche AB, Yates NE, Todd AD (2006) The recovery over several seasons of N-15-labelled fertilizer applied to Miscanthus x giganteus ranging from 1 to 3 years old. *Biomass & Bioenergy*, **30**, 125–133.

Dahms H, Mayr S, Birkhofer K, Chauvat M, Melnichnova E, Wolters V, Dauber J (2010) Contrasting diversity patterns of epigeic arthropods between grasslands of high and low agronomic potential. *Basic and Applied Ecology*, **11**, 6–14.

Dauber J, Jones MB, Stout JC (2010) The impact of biomass crop cultivation on temperate biodiversity. *Global Change Biology Bioenergy*, **2**, 289–309.

Davis SC, Parton WJ, Del Grosso SJ, Keough C, Marx E, Adler PR, DeLucia EH (2012) Impact of second-generation biofuel agriculture on greenhouse-gas emissions in the corn-growing regions of the US. *Frontiers in Ecology and the Environment*, **10**, 69–74.

Diaz RJ (2001) Overview of hypoxia around the world. *Journal of environmental quality*, **30**, 275–281.

Diaz RJ, Rosenberg R (1995) Marine benthic hypoxia: a review of its ecological effects and the behavioural responses of benthic macrofauna. *Oceanography and marine biology. An annual review*, **33**, 245–03.

Dimitriou I, Mola-Yudego B, Aronsson P (2012) Impact of Willow Short Rotation Coppice on Water Quality. *Bioenergy Research*, **5**, 537–545.

Don A, Osborne B, Hastings A et al. (2012) Land-use change to bioenergy production in Europe: implications for the greenhouse gas balance and soil carbon. *GCB Bioenergy*, **4**, 372–391.

Donnelly A, Styles D, Fitzgerald J, Finnan J (2011) A proposed framework for determining the environmental impact of replacing agricultural grassland with Miscanthus in Ireland. *Global Change Biology Bioenergy*, **3**, 247–263.

Dowell RC, Gibbins D, Rhoads JL, Pallardy SG (2009) Biomass production physiology and soil carbon dynamics in short-rotation-grown Populus deltoides and P. deltoides × P. nigra hybrids. *Forest Ecology and Management*, **257**, 134–142.

Drewer J, Finch JW, Lloyd CR, Baggs EM, Skiba U (2012) How do soil emissions of N2O, CH4 and CO2 from perennial bioenergy crops differ from arable annual crops? *Global Change Biology Bioenergy*, **4**, 408–419.

Eller ASD, Sekimoto K, Gilman JB et al. (2011) Volatile organic compound emissions from switchgrass cultivars used as biofuel crops. *Atmospheric Environment*, **45**, 3333–3337.

Evers BJ, Blanco-Canqui H, Staggenborg SA, Tatarko J (2013) Dedicated Bioenergy Crop Impacts on Soil Wind Erodibility and Organic Carbon in Kansas. *Agronomy Journal*, **105**, 1271.

Finch JW, Riche AB (2010) Interception losses from Miscanthus at a site in south-east England—an application of the Gash model. *Hydrological Processes*, **24**, 2594–2600.

Findlater KM, Kandlikar M (2011) Land use and second-generation biofuel feedstocks: The unconsidered impacts of Jatropha biodiesel in Rajasthan, India. *Energy Policy*, **39**, 3404–3413.

Fletcher RJ, Robertson BA, Evans J, Doran PJ, Alavalapati JR, Schemske DW (2011) Biodiversity conservation in the era of biofuels: risks and opportunities. *Frontiers in Ecology and the Environment*, **9**, 161–168.

Gardiner MA, Tuell JK, Isaacs R, Gibbs J, Ascher JS, Landis DA (2010) Implications of Three Biofuel Crops for Beneficial Arthropods in Agricultural Landscapes. *Bioenergy Research*, **3**, 6–19.

Garg KK, Karlberg L, Wani SP, Berndes G (2011) Jatropha production on wastelands in India: opportunities and trade-offs for soil and water management at the watershed scale. *Biofuels Bioproducts & Biorefining-Biofpr*, **5**, 410–430.

Gelfand I, Zenone T, Jasrotia P, Chen JQ, Hamilton SK, Robertson GP (2011) Carbon debt of Conservation Reserve Program (CRP) grasslands converted to bioenergy production. *Proceedings of the National Academy of Sciences of the United States of America*, **108**, 13864–13869.

German L, Schoneveld GC, Gumbo D (2011) The Local Social and Environmental Impacts of Smallholder-Based Biofuel Investments in Zambia. *Ecology and Society*, **16**.

Gonzalez-Garcia S, Mola-Yudego B, Dimitriou I, Aronsson P, Murphy R (2012) Environmental assessment of energy production based on long term commercial willow plantations in Sweden. *Science of the Total Environment*, **421**, 210–219.

Goolsby DA, Battaglin WA (2000) Nitrogen in the Mississippi Basin-estimating sources and predicting flux to the Gulf of Mexico. *USGS Fact Sheet*, 135–00.

Haney RL, Kiniry JR, Johnson MVV (2010) Soil microbial activity under different grass species: Underground impacts of biofuel cropping. *Agriculture Ecosystems & Environment*, **139**, 754–758.

Hickman GC, Vanloocke A, Dohleman FG, Bernacchi CJ (2010) A comparison of canopy evapotranspiration for maize and two perennial grasses identified as potential bioenergy crops. *Global Change Biology Bioenergy*, **2**, 157–168.

Hillier J, Whittaker C, Dailey G et al. (2009) Greenhouse gas emissions from four bioenergy crops in England and Wales: Integrating spatial estimates of yield and soil carbon balance in life cycle analyses. *Global Change Biology Bioenergy*, **1**, 267–281.

Holland RA, Eigenbrod F, Muggeridge A, Brown G, Clarke D, Taylor G (2015) A synthesis of the ecosystem services impact of second generation bioenergy production. *Renewable and Sustainable Energy Reviews*. DOI: 10.1016/j.rser.2015.02.003

Holzschuh A, Steffan-Dewenter I, Kleijn D, Tscharntke T (2007) Diversity of flower-visiting bees in cereal fields: effects of farming system, landscape composition and regional context. *Journal of Applied Ecology*, **44**, 41–49.

Kang S, Post WM, Nichols JA, Wang D, West TO, Bandaru V, Izaurralde RC (2013) Marginal Lands: Concept, Assessment and Management. *Journal of Agricultural Science*, **5**.

Keith AM, Rowe RL, Parmar K, Perks MP, Mackie E, Dondini M, McNamara NP (2014) Implications of land use change to Short Rotation Forestry in Great Britain for soil and biomass carbon. *GCB Bioenergy*.

Kirilenko AP, Sedjo RA (2007) Climate change impacts on forestry. *Proceedings of the National Academy of Sciences of the United States of America*, **104**, 19697–19702.

Klein AM, Vaissiere BE, Cane JH, Steffan-Dewenter I, Cunningham SA, Kremen C, Tscharntke T (2007) Importance of pollinators in changing landscapes for world crops. *Proc Biol Sci*, **274**, 303–13.

Kocoloski M, Michael Griffin W, Scott Matthews H (2011) Impacts of facility size and location decisions on ethanol production cost. *Energy Policy*, **39**, 47–56.

Kremen C, Williams NM, Aizen MA et al. (2007) Pollination and other ecosystem services produced by mobile organisms: a conceptual framework for the effects of land-use change. *Ecology Letters*, **10**, 299–314.

Landis DA, Werling BP (2010) Arthropods and biofuel production systems in North America. *Insect Science*, **17**, 220–236.

Lattimore B, Smith CT, Titus BD, Stupak I, Egnell G (2009) Environmental factors in woodfuel production: Opportunities, risks, and criteria and indicators for sustainable practices. *Biomass & Bioenergy*, **33**, 1321–1342.

Le PVV, Kumar P, Drewry DT (2011) Implications for the hydrologic cycle under climate change due to the expansion of bioenergy crops in the Midwestern United States. *Proceedings of the National Academy of Sciences of the United States of America*, **108**, 15085–15090.

Love BJ, Nejadhashemi AP (2011) Water quality impact assessment of large-scale biofuel crops expansion in agricultural regions of Michigan. *Biomass & Bioenergy*, **35**, 2200–2216.

Love BJ, Einheuser MD, Nejadhashemi AP (2011) Effects on aquatic and human health due to large scale bioenergy crop expansion. *Science of the Total Environment*, **409**, 3215–3229.

Lovett AA, Sunnenberg GM, Richter GM, Dailey AG, Riche AB, Karp A (2009) Land use implications of increased biomass production identified by GIS-based suitability and yield mapping for Miscanthus in England. *Bioenergy Research*, **2**, 17–28.

Ma Z, Wood CW, Bransby DI (2000) Soil management impacts on soil carbon sequestration by switchgrass. *Biomass & Bioenergy*, **18**, 469–477.

Monti A, Fazio S, Venturi G (2009) Cradle-to-farm gate life cycle assessment in perennial energy crops. *European Journal of Agronomy*, **31**, 77–84.

Myers JH, Higgins C, Kovacs E (1989) How Many Insect Species are Necessary for the Biological Control of Insects? *Environmental Entomology*, **18**, 541–547.

Ng TL, Eheart JW, Cai XM, Miguez F (2010) Modeling Miscanthus in the Soil and Water Assessment Tool (SWAT) to Simulate Its Water Quality Effects As a Bioenergy Crop. *Environmental Science & Technology*, **44**, 7138–7144.

Oliver RJ, Finch JW, Taylor G (2009) Second generation bioenergy crops and climate change: a review of the effects of elevated atmospheric CO2 and drought on water use and the implications for yield. *GCB Bioenergy*, **1**, 97–114.

Palmer MM, Forrester JA, Rothstein DE, Mladenoff DJ (2014) Conversion of open lands to short-rotation woody biomass crops: site variability affects nitrogen cycling and N2O fluxes in the US Northern Lake States. *Global Change Biology Bioenergy*, **6**, 450–464.

Pellegrino E, Di Bene C, Tozzini C, Bonari E (2011) Impact on soil quality of a 10-year-old short-rotation coppice poplar stand compared with intensive agricultural and uncultivated systems in a Mediterranean area. *Agriculture Ecosystems & Environment*, **140**, 245–254.

Power AG (2010) Ecosystem services and agriculture: tradeoffs and synergies. *Philosophical Transactions of the Royal Society B: Biological Sciences*, **365**, 2959–2971.

Raunikar R, Buongiorno J, Turner JA, Zhu SS (2010) Global outlook for wood and forests with the bioenergy demand implied by scenarios of the Intergovernmental Panel on Climate Change. *Forest Policy and Economics*, **12**, 48–56.

Rittenburg RA, Kummel M, Perramond EP (2011) The local climate-development nexus: Jatropha and smallholder adaptation in Tamil Nadu, India. *Climate and Development*, **3**, 328–343.

Robertson BA, Porter C, Landis DA, Schemske DW (2012) Agroenergy Crops Influence the Diversity, Biomass, and Guild Structure of Terrestrial Arthropod Communities. *Bioenergy Research*, **5**, 179–188.

Rowe RL, Street NR, Taylor G (2009) Identifying potential environmental impacts of large-scale deployment of dedicated bioenergy crops in the UK. *Renewable and Sustainable Energy Reviews*, **13**, 271–290.

Rowe RL, Hanley ME, Goulson D, Clarke DJ, Doncaster CP, Taylor G (2011) Potential benefits of commercial willow Short Rotation Coppice (SRC) for farm-scale plant and invertebrate communities in the agri-environment. *Biomass & Bioenergy*, **35**, 325–336.

Ruiz-Valdiviezo VM, Luna-Guido M, Galzy A, Gutierrez-Miceli FA, Dendooven L (2010) Greenhouse gas emissions and C and N mineralization in soils of Chiapas (Mexico) amended with leaves of Jatropha curcas L. *Applied Soil Ecology*, **46**, 17–25.

Sage RB (1998) Short rotation coppice for energy: towards ecological guidelines. *Biomass and Bioenergy*, **15**, 39–47.

Sanderson MA, Adler PR (2008) Perennial forages as second generation bioenergy crops. *International Journal of Molecular Sciences*, **9**, 768–788.

Schmer MR, Liebig MA, Vogel KP, Mitchell RB (2011) Field-scale soil property changes under switchgrass managed for bioenergy. *GCB Bioenergy*, **3**, 439–448.

Schulze ED, Korner CI, Law BE, Haberl H, Luyssaert S (2012) Large-scale bioenergy from additional harvest of forest biomass is neither sustainable nor greenhouse gas neutral. *Global Change Biology Bioenergy*, **4**, 611–616.

Skutsch M, de los Rios E, Solis S et al. (2011) Jatropha in Mexico: environmental and social impacts of an incipient biofuel program. *Ecology and Society*, **16**, 11.

Stanley DA, Stout JC (2013) Quantifying the impacts of bioenergy crops on pollinating insect abundance and diversity: a field-scale evaluation reveals taxon-specific responses (ed Clough Y). *Journal of Applied Ecology*, **50**, 335–344.

Stone KC, Hunt PG, Cantrell KB, Ro KS (2010) The potential impacts of biomass feedstock production on water resource availability. *Bioresource Technology*, **101**, 2014–2025.

Syswerda SP, Basso B, Hamilton SK, Tausig JB, Robertson GP (2012) Long-term nitrate loss along an agricultural intensity gradient in the Upper Midwest USA. *Agriculture Ecosystems & Environment*, **149**, 10–19.

Thomson LJ, Hoffmann AA (2011) Pest management challenges for biofuel crop production. *Current Opinion in Environmental Sustainability*, **3**, 95–99.

Toma Y, Fernandez FG, Sato S et al. (2011) Carbon budget and methane and nitrous oxide emissions over the growing season in a Miscanthus sinensis grassland in Tomakomai, Hokkaido, Japan. *Global Change Biology Bioenergy*, **3**, 116–134.

UK NEA (2011) The UK National Ecosystem Assessment. Synthesis of the Key Findings. *UNEP-WCMC, Cambridge*.

Updegraff K, Baughman MJ, Taff SJ (2004) Environmental benefits of cropland conversion to hybrid poplar: economic and policy considerations. *Biomass & Bioenergy*, **27**, 411–428.

Vanloocke A, Bernacchi CJ, Twine TE (2010) The impacts of Miscanthus x giganteus production on the Midwest US hydrologic cycle. *Global Change Biology Bioenergy*, **2**, 180–191.

VanLoocke A, Twine TE, Zeri M, Bernacchi CJ (2012) A regional comparison of water use efficiency for miscanthus, switchgrass and maize. *Agricultural and Forest Meteorology*, **164**, 82–95.

Werling BP, Meehan TD, Gratton C, Landis DA (2011a) Influence of habitat and landscape perenniality on insect natural enemies in three candidate biofuel crops. *Biological Control*, **59**, 304–312.

Werling BP, Meehan TD, Robertson BA, Gratton C, Landis DA (2011b) Biocontrol potential varies with changes in biofuel-crop plant communities and landscape perenniality. *Global Change Biology Bioenergy*, **3**, 347–359.

Werling BP, Dickson TL, Isaacs R et al. (2014) Perennial grasslands enhance biodiversity and multiple ecosystem services in bioenergy landscapes. *Proceedings of the National Academy of Sciences*, **111**, 1652–1657.

Wilson HM, Cruse RM, Burras CL (2011) Perennial grass management impacts on runoff and sediment export from vegetated channels in pulse flow runoff events. *Biomass & Bioenergy*, **35**, 429–436.

Woli KP, David MB, Darmody RG, Mitchell CA, Smith CM (2010) Assessing the nitrous oxide mole fraction of soils from perennial biofuel and corn-soybean fields. *Agriculture Ecosystems & Environment*, **138**, 299–305.

Wu YP, Liu SG (2012) Impacts of biofuels production alternatives on water quantity and quality in the Iowa River Basin. *Biomass & Bioenergy*, **36**, 182–191.

Xue X, Luo Y, Zhou X, Sherry R, Jia X (2011) Climate warming increases soil erosion, carbon and nitrogen loss with biofuel feedstock harvest in tallgrass prairie. *GCB Bioenergy*, **3**, 198–207.

Zimmermann J, Dauber J, Jones MB (2012) Soil carbon sequestration during the establishment phase of Miscanthus x giganteus: a regional-scale study on commercial farms using C-13 natural abundance. *Global Change Biology Bioenergy*, **4**, 453–461.

Zimmermann J, Dondini M, Jones MB (2013) Assessing the impacts of the establishment of Miscanthus on soil organic carbon on two contrasting land-use types in Ireland. *European Journal of Soil Science*, **64**, 747–756.
