# Supplementary material for: Potential impacts on ecosystem services of land use transitions to second‐generation bioenergy crops in GB
Source: Glob Change Biol Bioenergy. 2015 Jun 8;8(2):317–33. doi: 10.1111/gcbb.12263 (PMC4974899; doi:10.1111/gcbb.12263)
Supplement: Supplementary file 2 — Table S3. Predicted land availability and SOC change per region of GB based on SOC estimates and planting scenarios per region. [file GCBB-8-317-s002.docx]

Table S3. Predicted land availability and SOC change per region of GB based on SOC estimates and planting scenarios per region

|  | ***Miscanthus*** | | | | **SRC** | | | |
| --- | --- | --- | --- | --- | --- | --- | --- | --- |
|  | **Base Planted** | | **2020s Planted** | | **Base Planted** | | **2020s Planted** | |
| **Region** | **Hectares** | **SOC Chg Tons** | **Hectares** | **SOC Chg Tons** | **Hectares** | **SOC Chg Tons** | **Hectares** | **SOC Chg Tons** |
| Highlands and Islands | 0 | 0 | 0 | 0 | 9 | 8 | 0 | 0 |
| North Eastern Scotland | 0 | 0 | 0 | 0 | 0 | 0 | 0 | 0 |
| Eastern Scotland | 0 | 0 | 0 | 0 | 126 | 218 | 0 | 0 |
| South Western Scotland | 30 | 57 | 43 | 82 | 52 | 105 | 0 | 0 |
| North East | 0 | 0 | 0 | 0 | 188 | 274 | 111 | 159 |
| North West | 33,857 | 57,533 | 63,797 | 110,900 | 75,630 | 165,174 | 75,603 | 166,282 |
| Yorkshire and the Humber | 557 | 1,268 | 1,120 | 2,479 | 1,634 | 4,277 | 1,451 | 3,900 |
| East Midlands | 136 | 317 | 2,033 | 4,410 | 36 | 36 | 181 | 205 |
| West Midlands | 3,076 | 6,401 | 95,886 | 159,354 | 724 | 1,437 | 27,251 | 34,835 |
| East of England | 114 | 264 | 203 | 456 | 0 | 0 | 0 | 0 |
| London | 0 | 0 | 0 | 0 | 0 | 0 | 0 | 0 |
| South East | 15,672 | 43,243 | 20,593 | 56,034 | 0 | 0 | 99 | 149 |
| South West | 85,110 | 211,373 | 94,146 | 233,449 | 2,526 | 5,313 | 605 | 961 |
| Wales North | 6,453 | 11,397 | 25,575 | 39,805 | 5,558 | 11,905 | 5,809 | 12,491 |
| Wales East | 2,777 | 5,158 | 8,339 | 14,874 | 910 | 1,187 | 917 | 976 |
| Wales West | 12,892 | 27,136 | 23,758 | 49,561 | 980 | 1,529 | 793 | 985 |
| Wales South | 8,497 | 21,758 | 14,770 | 36,747 | 34 | 78 | 50 | 115 |
| Total | 169,171 | 385,905 | 350,263 | 708,150 | 88,407 | 191,543 | 112,870 | 221,058 |
